# *Scleranthus perennis* L.: Towards Comprehensive Metabolite Profiling, Antioxidant and Enzyme Inhibitory Activity

**DOI:** 10.3390/plants15192918

**Published:** 2026-09-23

**Authors:** Reneta Gevrenova, Inci Kurt-Celep, Gokhan Zengin, Dimitrina Zheleva-Dimitrova

**Affiliations:** 1 Department of Pharmacognosy, Faculty of Pharmacy, Medical University, 1000 Sofia, Bulgaria; dzheleva@pharmfac.mu-sofia.bg; 2 Department of Pharmaceutical Biotechnology, Faculty of Pharmacy, Istanbul Okan University, Istanbul 34940, Turkey; inci.celep@okan.edu.tr; 3 Physiology and Biochemistry Research Laboratory, Department of Biology, Science Faculty, Selcuk University, Konya 42130, Turkey; biyologzengin@gmail.com

**Keywords:** *Scleranthus perennis*, *C-, O-*flavone glycosides, LC-MS, antioxidants, enzyme inhibitory potential

## Abstract

*Scleranthus perennis* L. is an Eurasian species from Caryophyllaceae family, Sclerantheae tribe. The work aimed at investigating the profiling of secondary metabolites in *S. perennis* aerial parts by means of ultra-high-performance liquid chromatography–high-resolution mass spectrometry (UHPLC–HRMS). The profile was delineated by phenolic compounds with a γ-pyrone core, including 49 *C*-glycosyl flavones *O*-glycosylated on the sugar of the *C*-glycosylation and their acetyl, galloyl, and methoxycinnamoyl esters alongside di-*C*-flavone glycosides. For the first time, a variety of maltol-glycosides was annotated together with phenolic acids and their glycosides. The total phenolic and flavonoid contents were 27.7 ± 0.4 mg gallic acid equivalents/g dry extract (de) and 31.6 ± 0.1 mg rutin equivalents/g de. Moderate ABTS radical scavenging activity (IC_50_: 1.21 mg/mL) and reducing power in CUPRAC (EC_50_: 1.40 mg/mL) and FRAP (EC_50_: 0.61mg/mL) assays were observed. Pronounced inhibitory activity towards *α*-glucoside (IC_50_: 1.03 mg/mL) and acetylcholinesterase (IC_50_: 1.45 mg/mL) was established. Our results are a prerequisite for further phytopharmacological and chemophenetic studies.

## 1. Introduction

*Scleranthus perennis* L. from Caryophyllaceae family is native to Europe, North Africa, and West Asia [1]. The species is a perennial plant with small flowers without petals and green sepals with apparent white margins 0.3–0.5 mm-wide near the tip (WorldFloraOnline).

The molecular phylogenetic studies strongly supported the placement of *Scleranthus* genus in Alsinoideae subfamily and Sclerantheae tribe [1,2]. The Sclerantheae tribe forms a plant clade in the analysis of Greenberg and Donoghue [2]. Sclerantheae includes the Hawaiian genus *Schiedea,* as well as *Geocarpon*, *Honckenya*, *Wilhelmsia*, *Spergularia* (*S. heldrechii*) and four *Stellaria* species. Moreover, recently, Dominican *Stellaria howardii*, *S. minutifolia,* and *S. ovata* were referred to *Maguirellaria* genus [3].

Several *C-, O-* and di-*C*-flavone glycosides have been reported in the *S. perennis* aerial parts of Polish origin, including 4′-acetoxychrysoeriol [2″-*O*-(4‴-acetoxylglucosyl)]-8-*C*-xyloside/arabinoside, (2″-*O*-glucosyl)-8-*C*-xylosides of 4′-acetoxychrysoeriol, 4′-acetoxyluteolin and 4′-methylluteolin, lucenin-1, 5, 7, 3′-rihydroxyflavone 6-*C*-glucosyl-8-*C*-glucoside and chrysoeriol 6-*C*-glucosyl-8-*C*-arabinoside, alongside the phenolic derivative apiopaeonoside [4,5]. In addition, a few di-*C*- and *C-*, *O*-flavone glycosides have been tentatively annotated in *S. perennis* extracts. Quantitative analysis of the isolated compounds revealed that 4′-acetoxychrysoeriol (2″-*O*-glucosyl)-8-*C*-xyloside (scleranthoside C) dominated the methanol extracts and ethylacetate fraction, being present at 6.29 mg/g and 59.1 mg/g extract, respectively [4]. Generally, ethylacetate fraction was the richest in total phenolic compounds (240 mg pyrogalol equivalents/g extract) and total phenolic acids (7.47 mg caffeic acid equivalents/g extract). Lucenin-1 (luteolin 6-*C*-xylosyl-8-*C*-glucoside) showed remarkable radical scavenging activity in the DPPH (409 mg Trolox equivalents (TE)/g) and ABTS (796 mg TE/g) assays, and reducing power in FRAP (45 mM Fe^2+^ equivalents/g) and CUPRAC (116 TE/g) tests. In the anti-collagenase inhibitory activity study, the most prominent flavonoids were 4′-acetoxychrysoeriol [2″-*O*-(4‴-acetoxylglucosyl)]-8-*C*-xyloside/arabinoside with IC_50_ values of 36.06 and 39.59 μM, compared with epigallocatechin gallate (34.32 μM) [4,5].

Owing to the fact that a few studies on the phytochemistry and biological activity of *Scleranthus* species have been performed [4,5,6] and the advances in the phytopharmacology of the Caryophyllaceae family [7,8], *S. perennis* is drawing scientific interest. As highlighted in a recent review article, the structural diversity of flavonoids established in the Caryophyllaceae family makes *Scleranthus* species worthy of investigation for secondary metabolite profiling and pharmacological activity estimation [7].

As part of our ongoing investigation of Caryophyllaceae species [9,10,11,12], we undertook an in-depth profiling of secondary metabolites in *S. perennis* aerial parts by means of ultra-high-performance liquid chromatography–quadrupole–Orbitrap high-resolution mass spectrometry (UHPLC–HRMS). In the study, antioxidant activity through different mechanisms and enzyme inhibitory potential towards key enzyme targets were evaluated.

## 2. Results and Discussion

*S. perennis* extract contains a highly complex mixture of 49 *C-, O-* and di-*C*-flavone glycosides alongside 14 maltol-glycosides. It is noteworthy that both classes of secondary metabolites include a γ-pyrones core. In addition, a variety of phenolic acids and their derivatives were identified. Altogether, 98 individual phenolics were annotated or identified in the extract. Regarding flavonoids, corresponding retention times, molecular formulas, MS/MS fragmentation patterns in positive (+ESI) and in negative (−ESI) ion mode, and mass measurement errors are depicted in Table 1. Among these compounds, a few flavonoids were annotated in either (+) ESI or (−) ESI, displaying reliable fragment ions consistent with the fragmentation mechanism of the corresponding group. Taking into account the mass spectrometry behavior of the phenolic compounds, maltol glycosides and coumarins were annotated in (+) ESI/MS, whereas phenolic acids and derivatives were annotated/dereplicated in (−) ESI/MS (Table 1). A semi-quantitative relative approach was performed, and the relative contents are expressed as % peak area of each compound relative to the total peak areas of the corresponding group of secondary metabolites and all metabolites. Extracted ion chromatograms are presented in Figures S1–S3. Identification confidence levels were according to Çiçek et al., 2024 [13], and were as follows: confirmed structure except for one or more stereochemical aspects (B); tentative identification matched with a standard compound, match of at least t_R_, MS and MS/MS with an actual authentic standard analyzed in parallel, preferably supported by other online data (C); tentative identification based on libraries, model compounds etc. (D), relatively reliable evidence (D1); relatively poor evidence (D2) [13].

### 2.1. Flavonoids

Compounds **2** and **3** shared the same [M−H]^−^ at *m*/*z* 579.135(6) and [M+H]^+^ at *m*/*z* 581.148(50), consistent with C_26_H_28_O_15_ (Table 1). Di-*C*-glycosides were suggested by the fragment ions at *m*/*z* 399.072 [(M−H)-120-60]^−^ and 369.061 [(M−H)-120-90]^−^ (**2**) resulting from the cross-ring cleavages of a hexose (X_0_) and a pentose (X_1_) residue ^0,2^X_0_^−^/^0,3^X_1_ and ^0,2^X_0_^−^/^0,2^X_1_, respectively (Table 1). This assumption was supported by the fragmentation pattern in (+) ESI-MS/MS affording ions at *m*/*z* 395.076 [(M+H)-90-60-2H_2_O]^+^and 311.055 [(M+H)-150-90-CH_2_O]^+^(**2**) (Table 1). The RDA ions at *m*/*z* 133.028 (^1,3^B^−^) and 137.023 (^0,2^B^+^) pointed to the aglycone luteolin. Thus, **2** and **3** were assigned as luteolin *C*-pentosyl-*C*-hexoside isomers. This assignment is in line with the previously isolated luteolin 6-C-xylosyl-8-C-glucoside from *S. perennis* [4]. In the same manner, compounds **5**, **8** and **15**with [M−H]^−^ at *m*/*z* 563.140 and [M+H]^+^ at *m*/*z* 565.155 yielded prominent fragment ions at *m*/*z* 383.077 [(M−H)-120-60]^−^ and 353.067 [(M−H)-120-90]^−^ (**5**) in (−) ESI-MS/MS suggesting di-*C*-glycoside. This structure was supported by the fragment ions at *m*/*z* 379.081 [(M+H)-90-60-2H_2_O]^+^ and 295.060 [(M+H)-150-90-CH_2_O]^+^ (**5**) in (+) ESI-MS/MS. The aglycone apigenin was identified by the RDA ions at *m*/*z* 117.033 (^1,3^B^−^) and 121.028 (^0,2^B^+^) (Table 1). The MS/MS spectra in both positive and negative ion mode are depicted in Figure 1A,B. Accordingly, compounds **5**, **8**, and **15** were ascribed to apigenin *C*-pentosyl-*C*-hexoside isomers. Likewise, the fragmentation patterns of **7**, **9**, and **10** were delineated by the transitions: *m*/*z* 593.151 [M−H]^−^→383.077 [(M−H)-120-90]^−^, 595.165 [M+H]^+^→409.091 [(M+H)-90-60-2H_2_O]^+^ and 595.165[M+H]^+^→325.070 [(M+H)-150-90-CH_2_O]^+^ (**7**). (Table 1). The aglycone methylluteolin was discernible by the RDA ions at *m*/*z* 151.039 (^0,2^B^+^), 149.060 (^1,3^B^+^) along with *m*/*z* 310.048 [(M+H)-180-90-CH_3_]^+^ (**7**). Thus, **7**, **9**, and **10** were assigned as methylluteolin *C*-pentosyl-*C*-hexoside isomers and could be related to chrysoeriol 6-*C*-glucosyl-8-*C*-arabinoside, previously isolated from *S. perennis* [4].

On the other hand, MS/MS of an isobar (**23**) of apigenin *C*-pentosyl-*C*-hexoside was acquired. The concomitant loss of the hexosyl moiety alongside a water molecule in (−) ESI-MS/MS resulted in the indicative fragment ion at *m*/*z* 383.077 [(M−H)-(162+18)]^−^ (Table 1). A *C*-bonded pentosyl residue was evident from the transition *m*/*z* 563.140 → 293.046 [(M−H)-(162+18)-90]^−^, as has been observed in 2″-*O*-hexosyl-*C*-pentosyl flavones [10,14]. This assumption was supported by fragmentation pattern in (+) ESI-MS/MS affording the abundant fragment ions at *m*/*z* 403.102 [(M+H)-162]^+^, 367.081 [(M+H)-162-2H_2_O]^+^ and 313.070 [(M+H)-162-90]^+^(Table 1). Accordingly, **23** was assigned as apigenin (2″-*O*-hexosyl)-*C*-pentoside. In the same manner, compound **19** was assigned to luteolin (2″-*O*-hexosyl)-*C*-pentoside witnessed by the following transitions at *m*/*z* 579.135[M−H]^−^→399.072 [(M−H)-(162+18)]^−^→309.041[(M−H)-(162+18)-90]^−^ in (−) ESI-MS/MS (Table 1). This assignment was corroborated by the transitions at *m*/*z* 581.150 [M+H]^+^→419.097 [(M+H)-162]^+^→ 401.086 [(M+H)-162-H_2_O]^+^ and 581.150 [M+H]^+^→329.065 [(M+H)-162-90]^+^ in (+) ESI-MS/MS. Likewise, compound **28** afforded abundant fragment ions at *m*/*z* 413.088 [(M−H)-(162+H_2_O)]^−^ and 323.056 [(M−H)-(162+18)-90]^−^, together with *m*/*z* 397.091 [(M+H)-162-2H_2_O]^+^ and 343.081 [(M+H)-162-90]^+^ (Figure 2A,B). This fragmentation pattern was consistent with the structure of methylluteolin (2″-*O*-hexosyl)-*C*-pentoside.

A series of acetylated derivatives of *C-, O-*glycosylflavones was annotated. Two compounds, **24** and **31**, shared the same [M−H]^−^ at *m*/*z* 621.146 and [M+H]^+^ at *m*/*z* 623.160, consistent with C_28_H_30_O_16_ (Table 1). An acetyl functional group and a 2″-*O*-hexosyl moiety in the *C*-bonded pentose was evidenced by the prominent transitions: *m*/*z* 621.146→399.071 [(M−H)-(162+18)-42]^−^, 621.146→327.051 [(M−H)-(162+90)-42]^−^→309.051 [(M−H)-(162+18+90)-42]^−^ (**24**). Furthermore, fragment ions at *m*/*z* 461.107 [(M+H)-162]^+^, 383.075 [(M+H)-(162+18)-60]^+^ and 299.055 [(M+H)-(162+18)-90-3H_2_O]^+^ pointed on the acetylation of the aglycone luteolin (*m*/*z* 329.065→287.055), as has been observed in 4′-acetylluteolin (2″-*O*-glucosyl)-8-*C*-xyloside, previously isolated from *S. perennis* [5].

In the same way, two isomers (**26** and **36**) were assigned as acetylapigenin (2″-*O*-hexosyl)-*C*-pentoside, witnessed by the fragment ions at *m*/*z* 367.081 [(M+H)-(162+18)-60]^+^, 313.070 [(M+H)-(162+90)-42]^+^, and 271.060 [Apigenin+H]^+^ (C_15_H_11_O_5_) alongside 425.088 [(M−H)-(162+18)]^−^ and 293.046 [(M−H)-(162+18)-90-42]^−^ (**36**) (Table 1). On the other hand, the isomer **29** of the aforementioned compounds afforded prominent fragment ions at *m*/*z* 403.102 [(M+H)-162-42)]^+^ and 367.081 [(M+H)-(162+18)-42-H_2_O]^+^ along with 383.077 [(M−H)-(162+18)-42]^−^ and 293.046 [(M−H)-(162+18)-90-42]^−^, suggesting acetylation of the hexosyl residue. Accordingly, **29** was ascribed to apigenin (2″-*O*-acetylhexosyl)-*C*-pentoside.

Compounds **34**, **35** and **39** shared the same [M−H]^−^ at *m*/*z* 635.162 and [M+H]^+^ at *m*/*z* 637.175(6) (consistent with C_29_H_32_O_16_), indicating 14 Da higher than **24** and **31** (Table 1). The annotation of methylluteolin was evidenced by the typical loss of methyl group in the transitions: *m*/*z* 313.070 [(M+H)-162-90-42-30]^+^→298.047 (**34**), 343.081[(M+H)-162-90-42]^+^→328.058 (**35**) and 301.070 [methylluteolin+H]^+^→286.047 (**39**). Compound **34** and **39** were assigned as acetyl-methylluteolin-(2″-*O*-hexosyl)-*C*-pentoside isomers witnessed by the prominent fragment ions at *m*/*z* 397.091[(M+H)-(162+18)-60]^+^ and 313.070 [(M+H)-162-90-42-30]^+^ (**34**) together with 323.056 [(M−H)-(162+18)-90-42]^−^(Figure 2C,D). This assignment is in line with the previously isolated *S. perennis C-, O-*flavonoid 4′-acetylchrysoeriol (2″-*O*-glucosyl)-8-*C*-xyloside [5]. Methylluteolin (2″-*O*-acetylhexosyl)-*C*-pentoside (**35**) was evidenced by the ions at *m*/*z* 433.113 [(M+H)-162-42]^+^ and 343.081 [(M+H)-162-42-90]^+^ alongside 413.088 [(M−H)-(162+18)-42]^−^.

The fragmentation patterns of **33** and **42** with [M+H]^+^ at *m*/*z* 649.175(6) (C_30_H_33_O_16_) were closely related to those of **26** and **36**, except for the appearance of an additional acetyl group, evidenced by the fragment ions at *m*/*z* 445.112 [(M+H)-162-42]^+^ and 367.081 [(M+H)-(162+18)-42-60]^+^ (**33**), indicating an acetylated hexosyl residue (Figure 1C). This assumption was corroborated by the abundant ions at *m*/*z* 425.088 [(M−H)-(162+18)-42]^−^ and 293.046 [(M−H)-(162+18)-2×42-90]^−^ in (−) ESI-MS/MS (Figure 1D). Accordingly, **33** and **42** were assigned as acetylapigenin (2″-*O*-acetylhexosyl)-*C*-pentoside. Likewise, the fragmentation pattern of their isobar **37** was related to compound **29**, and a diacetylated hexosyl moiety was evidenced by the following transitions: 649.176→403.102 [(M+H)-(162+2×42)]^+^ and 647.162→383.077[(M−H)-(162+18)-2×42]^−^. Furthermore, an additional acetyl group in the hexosyl residue was evidenced in **40** and **50** in comparison with **33** and **42** by the prominent ions at *m*/*z* 313.070 [(M+H)-162-3×42-90)]^+^ and 293.046 [(M−H)-(162+18)-3×42-90]^−^ (**50**) (Table 1). Consequently, they were assigned as acetylapigenin [2″-*O*-(diacetylhexosyl)]-*C*-pentoside isomers.

Compounds **38**, **41**, **43**, and **48** afforded the same [M−H]^−^ at *m*/*z* 677.172 [M−H]^−^ and [M+H]^+^ at *m*/*z* 679.186 (consistent with molecular formula C_31_H_34_O_17_), indicating 42 Da higher than **34**, **35**, and **39** (Table 1). Taking into consideration the following transitions: 677.172→455.098 [(M−H)-(162+18)-42)]^−^→413.087 [(M−H)-(162+18)-2×42)]^−^(**38**) and 679.186→397.091 [(M+H)-(162+18)-2×42-18)]^+^, **38**, **41** and **43** could be annotated as methylluteolin [2″-*O*-(diacetyl hexosyl)]-*C*-pentoside isomers. On the other hand, **48** afforded fragment ions at *m*/*z* 455.098 [(M−H)-(162+18)-42)]^−^ and 323.056 [(M−H)-(162+18)-2×42-90)]^−^, indicating both an acetylated hexosyl residue and the aglycone. This assignment was corroborated by the ions at *m*/*z* 397.091 [(M+H)-(162+18)-42-60]^+^ and 343.081 [(M+H)-162-2×42-90]^+^ (Table 1). Compound **48** was ascribed to acetyl-methylluteolin-(2″-*O*-acetylhexosyl)-*C*-pentoside and could be related to 4′-acetylchrysoeriol [2″-*O*-(4‴-acetyl-glucosyl)]-8-*C*-xyloside/arabinoside, previously isolated from *S. perennis* [4,5]. In the same way, compounds **45** and **55** gave [M−H]^−^ at *m*/*z* 719.183 and [M+H]^+^ at *m*/*z* 721.196 (consistent with molecular formula C_33_H_36_O_18_), indicating 42 Da higher than **48** and a supplementary acetyl group bonded to the hexosyl residue (Table 1). Diacetylated hexosyl moety was dedicated from the following transitions: *m*/*z* 719.184→455.100 [(M−H)-(162+18)-2×42)]^−^→323.056 [(M−H)-(162+18)-3×42-90)]^−^ alongside *m*/*z* 397.091 [(M+H)-(162+18)-2×42-60]^+^ and 343.081 [(M+H)-162-3×42-90]^+^ (**55**). Thus, **45** and **55** were assigned as acetyl-methylluteolin [2″-*O*-(diacetylhexosyl)]-*C*-pentoside isomers. Likewise, a triacetylated hexosyl moiety was evidenced in **57**, **59**, and **61** by transitions at *m*/*z* 761.194→455.098 [(M−H)-(162+18)-3×42)]^−^→323.056 [(M−H)-(162+18)-4×42-90)]^−^(**61**) (Table 1, Figure 2E). Accordingly, the abovementioned compounds were related to acetyl-methylluteolin [2″-*O*-(triacetylhexosyl)]-*C*-pentoside isomers.

Compounds **44**, **46**, and **51** shared the same precursor ions at *m*/*z* 783.214 [M−H]^−^ and *m*/*z* 785.228 [M+H]^+^ (consistent with molecular formula C_38_H_40_O_18_) (Table 1). The concomitant losses of a methoxycinnamic acid (MecinOH) (C_10_H_10_O_3_) and a hexose unit (C_6_H_12_O_6_) were evidenced by the fragment ions at *m*/*z* 425.088 [(M−H)-(162+18)-178]^−^ supported by *m*/*z* 445.111 [(M+H)-162-178]^+^ and 341.123 [Mecinhexoside+H)]^+^ (**51**) (Figure 1E). Additionally, the MecinOH was deduced from the ions at *m*/*z* 179.070 [MecinOH+H]^+^, 164.046 [(MecinOH+H)-CH_3_]^+^, 151.075 [(MecinOH+H)-CO]^+^ and 137.060 [(MecinOH+H)-C_2_H_2_O]^+^. Prominent ions at *m*/*z* 293.046 [(M−H)-(162+18)-178-90-42]^−^ and 313.070 [(M+H)-162-178-42-90]^+^ pointed on the aglycone acetylapigenin. Accordingly, **44**, **46**, and **51** were assigned as acetylapigenin [2″-*O*-(methoxycinnamoylhexosyl)]-*C*-pentoside isomers. In the same manner, compounds **58**, **60** and **62** yielded prominent ions at *m*/*z* 455.098 [(M−H)-(162+18)-178-42]^−^, indicating a loss of both the acetyl group and MecinOH (Table 1, Figure 2F). Taking into consideration the abundant ions at *m*/*z* 323.056 (see **48**), the aforementioned compounds were ascribed to acetyl-methylluteolin [2″-*O*-(methoxycinnamoyl)-acetylhexosyl)]-*C*-pentoside isomers.

Compounds **47**, **54**, and **56** yielded the same [M+H]^+^ at *m*/*z* 777.187 (consistent with molecular formula C_35_H_36_O_20_) (Table 1). The galloylhexosyl residue was deduced from the loss of 332.074 Da (C_13_H_16_O_10_) at *m*/*z* 445.113[(M+H)-162-170]^+^ (Figure 1F) [15]. In addition, the galloyl residue (GA) was supported by the fragment ions at *m*/*z* 171.029 [GA+H]^+^, 127.039 [(GA+H)-CO_2_]^+^, and 109.029 [(GA+H)-CO_2_-H_2_O]^+^. The aglycone was assigned as acetylapigenin, witnessed by the ions at *m*/*z* 313.070 [(M+H)-162-170-90-42]^+^ and 271.059 [apigenin+H)]^+^. Thus, **47**, **54**, and **56** were annotated as acetylapigenin [2″-*O*-(galloylhexosyl)]-*C*-pentoside isomers.

Compounds 6, 13, 16, 17, 20, 21, 25, 30, 32, and 53 were identified as homoorientin, rutin, hyperoside, luteolin 7-*O*-glucoside, kaempferol 3-*O*-rutinoside, apigenin 7-*O*-glucoside, isovitexin, vitexin, and luteolin, respectively, by comparison of retention times and fragmentation patterns of reference standards (Table 1). Among the group of flavonoids, the profile was dominated by acetyl-methylluteolin (2″-*O*-acetylhexosyl)-*C*-pentoside (**28**) (15.29%), acetyl-methylluteolin [2″-*O*-(diacetyl-hexosyl)]-*C*-pentoside 2 (**55**) (12.36%), acetyl-methylluteolin (2″-*O*-hexosyl)-*C*-pentoside 2 (**39**) (7.93%), and acetylapigenin [2″-*O*-(galloylhexosyl)]-C-pentoside 3 (**56**) (7.17%) (Table 1). Overall, flavonoids were found to be the major group of secondary metabolites in the tested extract, accounting for 66.6% of the assayed compounds.

### 2.2. Maltol Glycosides—Fragmentation Pathways and Chemophenetic Relevance

Maltol glycosides refer to a special class of γ-pyrones consisting of a maltol (3-hydroxy-2-methyl-γ-pyrone) skeleton linked to a glycosidic moiety, sometimes possessing an acyl group [16]. In (+) ESI-MS/MS, the precursor ions [M+H]^+^ loss glycosidic/acylglycosidic part and afforded a base peak at *m*/*z* 127.039 [maltol+H]^+^ (C_6_H_7_O_3_) (Table 1). Hence, the characteristic neutral losses at *m*/*z* 109.029 [maltol+H-H_2_O]^+^, 97.029 [maltol+H-CH_2_O]^+^ and 85.029 [maltol+H-C_2_H_2_O]^+^ were employed to recover maltol residue in each derivative. Maltol glycosides typically cleave at C-3 and loss glycosidic/acylglycosidic part. Exemplified by the isobaric pairs **64**/**65** and **63**/**67** and compound **66**, maltol hexosides, dihexosides, and pentosylhexoside were annotated, respectively, as witnessed by the losses of 162 Da (Hex), 324 Da (2 Hex), and 294 Da (Pent+Hex) (Table 1, Figure 3A). The MS/MS spectra of **68**/**69** at *m*/*z* 375.091 (C_15_H_18_O_11_) and **72**/**73** at *m*/*z* 461.092 (C_18_H_20_O_14_) were acquired (Table 1). Malonylhexosyl and dimalonylhexosyl moieties were assigned by the prominent losses of C_9_H_12_O_8_ (248.052 Da) (**68**/**69**) and C_12_H_14_O_11_ (334.053 Da) (**72**/**73**), respectively. An additional hexose unit was evidenced in **70** in comparison with **68**/**69** by the following transitions: *m*/*z* 537.144 [M+H]^+^→375.092 [M+H-C_6_H_10_O_5_]^+^→127.039 [M+H-C_6_H_10_O_5_-C_9_H_12_O_8_]^+^. Thus, **70** was assigned as maltol (malonylhexosyl)-hexoside (Table 1). Concerning **71**, the fragmentation pathway was delineated by the subsequent losses of a hydroxymethylglutaroyl residue (144.042 Da) at *m*/*z* 289.091 [M+H-C_6_H_8_O_4_]^+^ and a hexosyl unit (162.052 Da)at *m*/*z* 127.039 [M+H-C_6_H_8_O_4_-C_6_H_10_O_5_]^+^ (Figure 3C). Accordingly, **71** was ascribed to maltol (hydroxymethylglutaroyl)-hexoside, as has been observed in *Herniaria glabra* (Caryophyllaceae), where licoagroside B (maltol 3-*O*-[6-*O*-(3-hydroxy-3-methylglutaroyl)]-β-D-glucopyranoside) has been identified [17]. In the same manner, both malonyl and methoxycinnamoyl groups were deduced in **75**/**76** by the transitions as follows: *m*/*z* 553.154 [M+H]^+^→305.102 [M+H-C_9_H_12_O_8_]^+^→127.039 [M+H-C_9_H_12_O_8_-C_10_H_10_O_3_]^+.^ Thus, **75**/**76** were annotated as maltol [(malonyl)-methoxycinnamoyl]-hexoside isomers. An additional hexosyl unit was evidenced in **74** ([M+H]^+^ at *m*/*z* 715.205) in comparison with the aforementioned compounds, and its structure was ascribed to maltol [(malonylhexosyl)-methoxycinnamoyl]-hexoside (Figure 3D). All annotated maltol glycosides were represented as level D1 tentative structures.

Maltol glucopyranoside and maltol 3-{2′-[β-D-glucoside-(1′′′-3′′′)-β-D-glucoside]-propionyloxy-4′-methoxy-phenyl have been previously isolated from the *S. uncinatus* [6,18]. Three maltol glycosides, including maltol 3-[6′-*O*-malonyl-(β-D-glucopyranosyl)], maltol 3-{[4′-*O*-(3-hydroxy-3-methylglutaroyl]-[β-D-glucopyranosyl]} and licoagroside B (maltol 3-[6′-*O*-(3-hydroxy-3-methylglutaroyl)]-β-D-glucopyranoside, have been isolated from *Herniaria glabra* entire plant [17]. The aforementioned compounds contain a malonyl or hydroxymethylglutaroyl moiety connected to maltol through β-D-glucose. In this study, 14 maltol glycosides were annotated, including two maltol hexosides and their malonyl, dimalonyl, hydroxymethylglutaroyl, and malonyl-methoxycinnamoyl esters alongside two maltol dihexosides and their malonyl and malonyl-methoxycinnamoyl esters. Except for maltol hexoside, 13 maltol glycosides and acylglycosides are reported in the genus *Scleranthus* for the first time. Two of them were previously isolated from *H. glabra*. In addition, licoagroside B has been identified in *H. polygama* and *H. incana* [19]. Thus, maltol glycosides could be assumed to be a chemophenetic marker of *S. perennis*.

It is worth noting that maltol glycosides reached up to 25.5% of the annotated compounds, with a prevalence of maltol [(malonylhexosyl)-methoxycinnamoyl]-hexoside (**74**) (46.42%), maltol [(malonyl)-methoxycinnamoyl]-hexoside 1 (**75**) (12.91%), and maltol hexoside 1 (**64**) (10.94%) (Table 1).

### 2.3. Phenolic Acids and Derivatives

A series of hydroxybenzoic acid-hexosides (**77**, **78**, **79**, **81**, and **86**) and hydroxycinnamic acid-hexosides (**82**, **84**, **87**, and **90**) were annotated in the assayed extract. On the other hand, in (−) ESI-MS/MS compound **91** with [M−H]^−^at *m*/*z* 357.119 (C_16_H_21_O_8_) yielded prominent fragment ions at *m*/*z* 195.065 [M−H-Hex]^−^ (C_10_H_11_O_4_), 151.075 [M−H-Hex-CO_2_]^−^, 136.052 [M−H-Hex-CO_2-_CH_3_]^−^ and 123.044 [M−H-Hex-C_3_H_4_O_2_]^−^, indicating methoxy-phenoxy-propanoic acid (GMPPA). Compound **91** was ascribed to (glucosyloxy-methoxyphenyl)-propanoic acid, as has been observed in *Herniaria polygama*, where 3-(2-glucosyloxy-4-methoxyphenyl)-propanoic acid was identified[19].

Protocatechuic acid (**80**), 4-hydroxybenzoic acid (**83**), 3-hydroxybenzoic acid (**85**), chlorogenic acid (**89**), caffeic acid (**92**), o-coumaric acid (**94**), 3, 5-dicaffeoylquinic acid (**95**) and salicylic acid (**96**) were unambiguously identified on the basis of comparison of retention times and fragmentation patterns with reference standards.

Among this group, herniarin (**98**) (38.79%) was found to be the major compound, followed by 3-hydroxybenzoic acid (**83**) (14.70%) and salicylic acid (**96**) (11.57%) (Table 1). Overall, the phenolic acids and coumarins accounted for 7.9% of the assayed compounds.

### 2.4. Antioxidant and Enzyme Inhibitory Potential

Spectrophotometric techniques were employed to evaluate the total phenolic and flavonoid contents, as well as the antioxidant and enzyme inhibitory activities, of the tested extract. The total phenolic and flavonoid contents in the extract were found to be 27.7 mg GAE/g and 31.6 mg RE/g, respectively (Table 2). These outcomes align with the UHPLC–HRMS analysis, which indicates the presence of a broad range of flavonoids in the extract, including *C-, O-,* and di-*C*-flavone glycosides. Similarly, Jakimiuk et al. [4] observed elevated levels of flavonoid glycosides in the fractions of *S. perennis*.

The concept of antioxidants encompasses several mechanisms, such as radical scavenging, reducing power, and chelation of transition metals. Consequently, no single universal method has been established to provide a complete antioxidant profile, and multiple assays are typically employed to better represent antioxidant capacity. In this context, we evaluated the antioxidant properties of the *S. perennis* extract using various chemical assays (Table 3). Specifically, DPPH and ABTS assays were used to determine radical scavenging activity, yielding IC_50_ values of 2.37 mg/mL and 1.21 mg/mL, respectively. However, Trolox exhibited promising radical scavenging abilities against these radicals (IC_50_ of 0.05 mg/mL in the DPPH and 0.07 mg/mL in the ABTS assay). Electron-donating capacity is a key antioxidant mechanism, with higher electron donation generally indicating stronger antioxidant activity. The CUPRAC and FRAP assays were applied to measure the electron-donating ability of the extract, and the EC_50_ values were 1.40 mg/mL and 0.61 mg/mL, respectively. The phosphomolybdenum assay, which is based on the reduction in Mo (VI) to Mo (V) by antioxidant compounds and is regarded as a measure of total antioxidant capacity, gave an EC_50_ value of 1.96 mg/mL, as presented in Table 3. Chelation of transition metals is another crucial antioxidant mechanism, as it can limit hydroxyl radical formation via the Fenton reaction. The extract demonstrated metal-chelating activity, with an IC_50_ value of 1.54 mg/mL. To the best of our knowledge, information regarding the antioxidant capacity of *S. perennis* is still scarce. The antioxidant effects observed in the present work are likely due to the presence of flavonoids, which are widely recognized for their antioxidant capacity [20,21,22].

Significant advances have been made in understanding the mechanisms of antioxidant action of the flavone C-glycosides [23,24,25]. Indeed, extracts enriched in flavone C-glycosides are drawing increased interest from phytochemists due to the carbon-carbon bond between the glycosyl group and the aglycone core, making them more stable during enzymatic or acid hydrolysis [26]. It has been reported that orientin showed higher radical scavenging activity against DPPH radicals (IC_50_ 9–15 μM) than homoorientin (IC_50_ 9–34 μM) and orientin 2″-xyloside (IC_50_ 14 μg/mL) [25]. In general, flavone C-glycosides markedly contribute to scavenging DPPH radicals in comparison with *O*-glycosides: IC_50_ of luteolin 7-*O*-glucoside was 8–55 μM, whereas IC_50_ of 7-*O*-rutinoside was 3.4 mg/mL. In another study, orientin and lucenin-1 (luteolin 6-C-xylosyl-8-C-glucoside) showed strong ABTS scavenging activity with IC_50_ values of 30.2 and 31.7 μM, respectively [26]. 4′-acetoxyluteolin (2″-*O*-glucosyl)-8-*C*-xyloside (scleranthoside A) and lucenin-1, isolated from *S. perennis*, showed high activity in DPPH and ABTS assays with Trolox equivalents (TE) values of more than 400 μM TE (DPPH) and more than 765 μM TE (ABTS), followed by chrysoeriol-6-*C*-glucosyl-8-*C*-arabinoside [4]. The aforementioned glycosides demonstrate high reducing power: 581 and 116 μM TE (CUPRAC), and 39.3 and 45.3 mM TE (FRAP). In the literature, homoorientin and lucenin-1 exhibited TEAC_CUPRAC_ 3.4 and 3.1 μM Trolox/μM [26]. Likewise, homoorientin, orientin, and lucenin-1 showed the highest ferric-reducing antioxidant activity in the FRAP assay with TEAC_FRAP_ 1.6, 1.31, and 1.3 μM Trolox/μM. Nevertheless, the luteolin *C*-glycosides had remarkable antioxidant activity in the iron-chelation assay, with homoorientin showing the highest value, TEAC 5.0 μM Trolox/μM. As shown by Ghozzi et al., luteolin and its C-glycosides exhibited higher antioxidant activity compared to apigenin *C*-glycosides [26]. This clearly indicates that the free hydroxyl groups in the B-ring play a key role in antioxidant properties. Taking into consideration the results of Jakimiuk et al., the presence of an acetyl group at C-4′ of luteolin influences the antioxidant capacity compared to the other compounds, especially their ability to scavenge free radicals [4]. On the other hand, the methyl group at C-3′ (chrysoeriol) in *C*-, *O*-glycosides also affects the activity, being significantly lower than flavonoids with a free hydroxyl group.

It is noteworthy that maltol glycoside licoagroside B has been studied for antioxidant activity in human blood plasma exposed to oxidative stress in vitro [17]. At 1.9 μM, it reduced oxidative damage to proteins and decreased lipid peroxidation.

The ability to inhibit specific enzymes is now a key part of tackling major global health problems like diabetes mellitus, obesity, and Alzheimer’s disease. In this context, numerous compounds have been chemically synthesized as enzyme inhibitors; however, many of these exhibit undesirable side effects, including gastrointestinal disturbances and toxicity. Consequently, there is a growing demand for natural, safe inhibitors in pharmaceutical applications [27]. In the present study, we investigated the enzyme inhibitory activities of *S. perennis* extracts against cholinesterase, amylase, glucosidase, tyrosinase, collagenase, elastase, and hyaluronidase (Table 4). The extract exhibited an inhibitory effect on AChE with an IC_50_ value of 1.45 mg/mL, while it was not active on BChE at test concentrations up to 5 mg/mL. IC_50_ value for tyrosinase inhibitory ability was also found to be 1.42 mg/mL. Antidiabetic ability was examined by amylase and glucosidase inhibition, and the results were 4.02 mg/mL and 1.03 mg/mL, respectively. The extract of *Scleranthus* exerted the strongest inhibitory activity across all enzymes, with IC_50_ values of 37.2 ± 2.0 µg/mL for collagenase, 39.8 ± 2.8 µg/mL for elastase, and 59.4 ± 5.5 µg/mL for hyaluronidase (Table 5). These findings indicate that *S. perennis* possesses the highest enzyme inhibitory capacity linked with global health problems. This observation for collagenase is consistent with a previous study [5] reporting that flavonoid C-glycosides isolated from *S. perennis* exhibit anti-collagenase activity within a similar range (IC_50_: 36.06–70.24 µM, equivalent to approximately 20–40 µg/mL), confirming the relevance of its phytochemical profile in enzyme inhibition. Based on this finding, the enzyme inhibitory activity observed may be attributed to the presence of flavonoids [28,29,30]. In a comparison of AChE inhibitory potential of the flavone C-monoglucosides, homoorientin and orientin showed the highest activity [24]. No correlation between AChE inhibitory activity and antioxidant activity of flavone C-glycosides was found.

In a comparative study of flavonoid aglycones and glycosides for tyrosinase inhibitory activity, flavones *C-, O-*glycosides scleranthoside A-D showed weak potential with IC_50_ > 500 μM, while flavones *C*-monoglycosides isoorinetin and vitexin were more potent inhibitors with IC_50_ of 132 and 133 μM [31]. However, *C*-glycosylation, methylation, and acetylation of the hydroxyl groups were emphasized as important structural characteristics in kinetic reactions and molecular docking scores.

In general, the glycosylation at *C*-6 and *C*-8 in ring A of flavones decreased the inhibitory potential towards α-glucosidase: luteolin—and apigenin—monoglucosides have IC_50_ values between 19.7 and 25.1 μg/mL [32]. An additional hydroxyl group in the B-ring might reduce enzyme inhibition, but acylation improved the effect [33]. C-, O-glycosylflavones exhibited a weak effect in comparison with the positive control, acarbose [34].

However, due to the complex nature of phytochemicals in *S. perennis* extract and their interactions, the observed inhibitory effects may be influenced by multiple components. Therefore, further studies are needed to isolate individual compounds and determine which of them are specifically responsible for the observed activities.

## 3. Materials and Methods

### 3.1. Plant Material

*Scleranthus perennis* aerial parts were collected from one population located in Tzraclevci (Dragoman District) (42.9503′ N 23.1052′ E, altitude 853 m a.s.l.), during the full flowering stage, in July 2024. The plant material was identified by Assoc. Prof. Reneta Gevrenova according to WorldFloraOnline [35]. A voucher specimen was deposited at the Herbarium Academiae Scientiarum Bulgariae (SOM 179539). Twenty individual plants with equal sun exposure were harvested. The aerial parts were taken from the apex of the plant to its bottom. Thus, the plant material corresponds to the branched stems (up to 20 cm long) with sessile leaves and sessile blossoms grouped in clusters together with their styliform bracts. Plant samples were dried at room temperature (20–22 °C) and 50% relative humidity until they reached a constant weight. All plant samples were pooled and comminuted with a grinder (Rohnson, R-942, 220–240 V, 50/60 Hz, 200 W, Prague, Czech Republic). The powdered plant material was stored in a cool, dry place until further examination.

### 3.2. Sample Extraction

Air-dried powdered aerial parts (50.1 g) were extracted with 80% MeOH (1:30 *w*/*v*) by ultrasound (100 kHz, ultrasound bath Biobase UC-20C, Jinan, China) for 15 min, three times, at room temperature. The methanol was evaporated in vacuo, and the water residue was lyophilized (lyophilizer Biobase BK-FD10P, Biobase, Jinan, China; −65 °C) to yield the crude extract (6.5 g). Then, the lyophilized extract was dissolved in 80% methanol (0.1 mg/mL), filtered through a 0.45 μm syringe filter, and subjected to UHPLC–HRMS analyses. The same extract was used for pharmacological tests.

### 3.3. Chemicals

Acetonitrile (for LC–MS), formic acid (for LC–MS), and methanol (for HPLC) were purchased from Honeywell (Charlotte, NC, USA). The reference standards were obtained from Extrasynthese (Genay, France) (for homoorientin, rutin, hyperoside, luteolin 7-*O*-glucoside, kaempferol 3-*O*-rutinoside, isorhamnetin 3-*O*-rutinoside, apigenin 7-*O*-glucoside, vitexin, isovitexin, luteolin, 3-hydroxybenzoic, 4-hydroxybenzoic, salicylic, O-coumaric, chlorogenic, and caffeic acids). 3,5-dicaffeoylquinic acid was previously isolated and identified in our laboratory [36].

2,2′-azino-bis(3-ethylbenzothiazoline-6-sulphonic acid (ABTS), 1,1-diphenyl-2-picrylhydrazyl (DPPH), gallic acid, rutin, electric eel acetylcholinester-ase (AChE) (type-VI-S, EC 3.1.1.7), horse serum butyrylcholinesterase (BChE) (EC 3.1.1.8), galantamine, acetylthiocholine iodide (ATChI), butyrylthiocholine chloride (BTChI), 5,5-dithio-bis(2-nitrobenzoic) acid (DTNB), tyrosinase (EC1.14.18.1, mush-room), glucosidase (EC. 3.2.1.20, from Saccharomyces cerevisiae), amylase (EC. 3.2.1.1, from porcine pancreas), sodium carbonate, Folin–Ciocalteu reagent, hydrochloric acid, sodium hydroxide, trolox, ethylenediaminetetraacetate (EDTA), neocuproine, cupric chloride, ammonium acetate, ferric chloride, 2,4,6-Tris(2-pyridyl)-s-triazine (TPTZ), ammonium molybdate, ferrozine, ferrous sulphate hexahydrate, kojic acid, and acarbose were purchased from Sigma-Aldrich (Darmstadt, Germany). All chemicals were of analytical grade.

### 3.4. UHPLC–HRMS

The UHPLC–HRMS analyses were performed as previously described [37] on a Q Exactive Plus mass spectrometer (ThermoFisher Scientific, Inc., Waltham, MA, USA) with a heated electrospray ionization (HESI-II) probe (ThermoScientific), in negative and positive ion modes within the m/z range from 150 to 1500. The other parameters were as follows: spray voltage 3.5 kV (+) and 2.5 kV (−); sheath gas flow rate 38; auxiliary gas flow rate 12; spare gas flow rate 0; capillary temperature 320 °C; probe heater temperature 320 °C; S-lens RF level 50; and scan mode: full MS (resolution 70,000) and MS/MS (17,500). The chromatographic separation was achieved on a reversed-phase column C18 (1.8 µm, 2.1 × 100 mm), and the temperature was 40 °C. The mobile phase consisted of 0.1% formic acid in water (A) and 0.1% formic acid in acetonitrile (B); the run time was 33 min; the flow rate was 0.3 mL/min. The gradient elution program used was as follows: 0–1 min, 0–5% B; 1–20 min, 5–30% B; 20–25 min, 30–50% B; 25–30 min, 50–70% B; 30–33 min, 70–95%; 33–34 min, 95–5% B. The injection volume was 1 µL, and the flow rate was 300 µL/min. Data were processed using Xcalibur 4.2 (Thermo Fisher Scientific, Waltham, MA, USA) instrument control/data handling software, and raw files were obtained. Other chromatographic conditions were previously described. Xcalibur 4.2 (Thermo Fisher Scientific, Waltham, MA, USA) software was used for data processing.

The obtained raw file was processed by MZmine 2.0 software using targeted feature detection focusing on precursor ions and corresponding retention times. The parameters intensity tolerance of 80%, *m*/*z* tolerance of 5 ppm, and t_R_ tolerance of 0.03 min were used to achieve peak area and export the MS1 list. The relative contents are expressed as % peak area of each compound to the total peak areas of the corresponding group secondary metabolites and all metabolites.

### 3.5. Assay for Total Phenolic and Flavonoid Contents

Total phenolics and flavonoids quantification was carried out according to the methods previously described [38]. Gallic acid (GA) and rutin (RE) served as standards in the assays, and the outcomes were reported as gallic acid equivalents (GAE) and rutin equivalents per gram of extract.

### 3.6. Assays for In Vitro Antioxidant Capacity

The antioxidant activity of the studied extract was evaluated [38]. The DPPH, ABTS radical scavenging, and metal chelating assays were evaluated by IC_50_ (concentration of the sample required to scavenge/chelate 50% of the radicals or metals). CUPRAC, FRAP, and phosphomolybdenum assays were expressed as EC_50_ (effective concentration at absorbance 0.5) values. Trolox and EDTA (for metal chelating assay) were used as positive controls. All experiments were conducted in triplicate. The experimental details are given in the Supplemental Materials.

### 3.7. Inhibitory Effects Against Some Key Enzymes

Enzyme inhibition experiments on the samples were conducted following established protocols [38,39,40]. Galanthamine (for AChE and BChE), kojic acid (for tyrosinase), acarbose (for amylase and glucosidase), EGCG (for collagenase and elastase), and tannic acid (for hyaluronidase) were used as positive controls. The results were explained by IC_50_ values. All experiments were conducted in triplicate. The experimental details are given in the Supplemental Materials.

## 4. Limitation of the Study

Due to the lack of reference materials, the annotation of each peak with a putative metabolite was performed. Conclusive identification cannot be carried out by means of liquid chromatography hyphenated to HRMS because not all isomers are available as reference standards. In the current study, taking into consideration the limited availability of standards of di-*C*- and *C-, O-*flavone glycosides, and maltol glycosides, the annotation of the majority of metabolites became challenging. Additionally, numerous *C-, O-*flavone glycosides and maltol glycosides cannot be related to compounds reported in *Scleranthus* genus and Caryophyllaceae family. Thus, new derivatives need NMR verification. The outcomes from the antioxidant and enzyme inhibitory activity of the studied extract are prerequisites for further in vivo pharmacological studies. On the other hand, such kind of experiments are essential in the context of bioactive natural product prioritization.

## 5. Conclusions

Herein, 98 secondary metabolites, including 49 *C*-glycosyl flavones *O*-glycosylated on the sugar of the *C*-glycosylation and di-*C*-glycosyl flavones of apigenin, luteolin and methylluteolin were annotated in the *S. perennis* methanol–aqueous extract. HRMS/MS fragmentation patterns of a series of acetyl, galloyl, and methoxycinnamoyl esters of *C-*and *O-*flavone glycosides were proposed. In-depth profiling revealed mainly phenolic compounds with a γ-pyrone core, including flavone-glycosides, flavonol-glycosides, and maltol-glycosides. They have significance for the observed radical scavenging activity, reducing power, and metal-chelating capacity. The results showed promising enzyme inhibitory potential against α-glucosidase and acetylcholinesterase. This study presents the first comprehensive UHPLC–HRMS metabolic profiling of *S. perennis* and reports maltol glycoside derivatives in this species for the first time. Thus, our investigation is a prerequisite for a chemophenetic study. The presented results argue for additional pharmacological experiments which bring a light on the pharmaceutical application of *S. perennis* aerial parts.

## Figures and Tables

**Figure 1:**
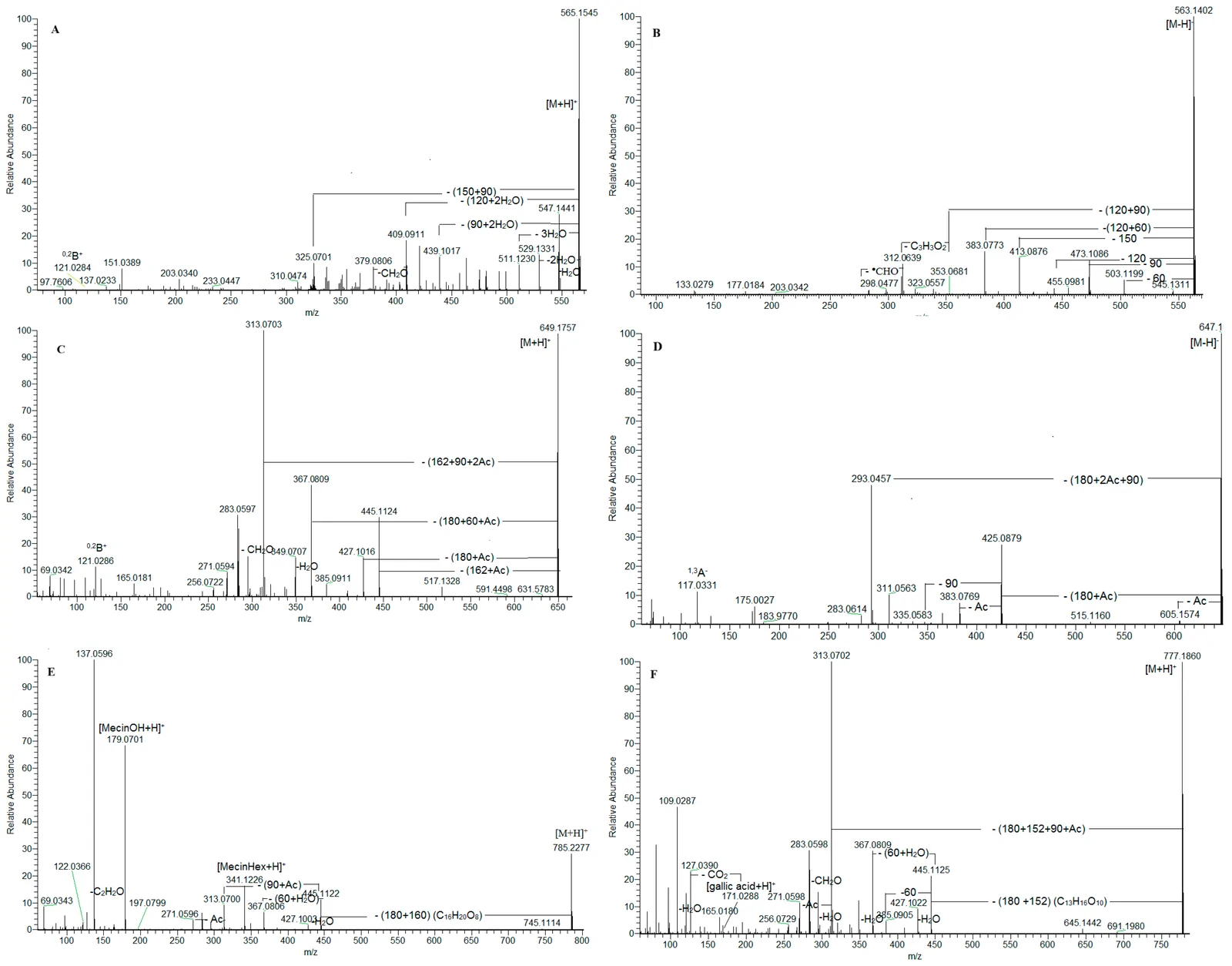
ESI-MS/MS spectra of apigenin di-*C*- and *C-, O-*glycosides: apigenin-*C*-pentoside-*C*-hexoside (**15**) in positive (**A**) and negative (**B**) ion mode; acetylapigenin-(2″-*O*-acetylhexosyl)-C-pentoside (**42**) in positive (**C**) and negative (**D**) ion mode; acetylapigenin-[2″-*O*-(methoxycinnamoylhexosyl)]-*C*-pentoside (**51**) in positive ion mode (**E**); acetylapigenin-[2″-*O*-(galloylhexosyl)]-*C*-pentoside (**56**) in positive ion mode (**F**).

**Figure 2:**
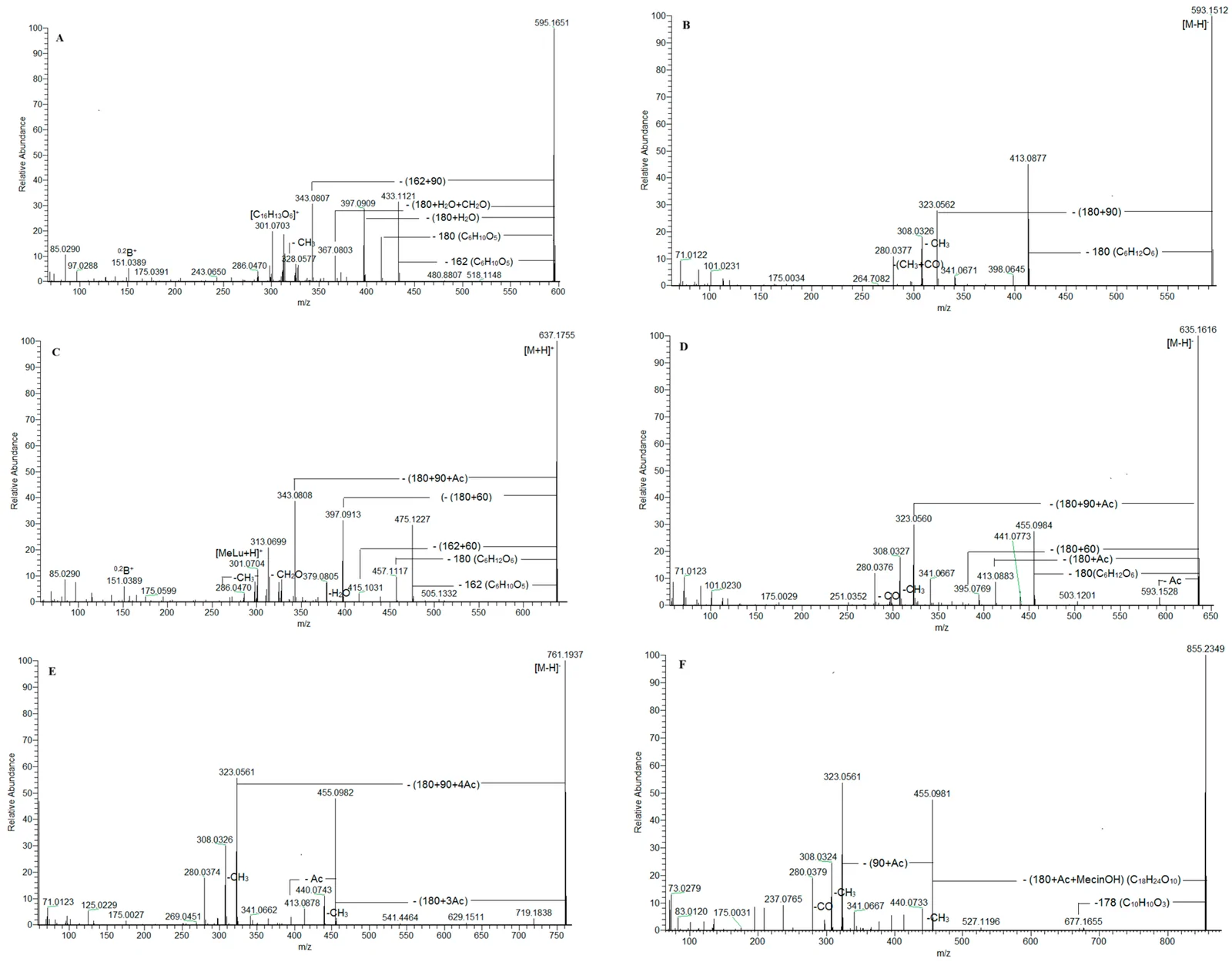
ESI-MS/MS spectra of methylluteolin *C-, O-*glycosides: methylluteolin-(2″-*O*-hexosyl)-*C*-pentoside (**28**) in positive (**A**) and negative (**B**) ion mode; acetyl-methylluteolin-(2″-*O*-hexosyl)-*C*-pentoside (**39**) in positive (**C**) and negative (**D**) ion mode; acetyl-methylluteolin-[2″-*O*-(triacetylhexosyl)]-C-pentoside (**59**) in negative ion mode (**E**); acetyl-methylluteolin-[2″-*O*-(methoxycinnamoyl-acetylhexosyl)]-*C*-pentoside (**60**) in negative ion mode (**F**).

**Figure 3:**
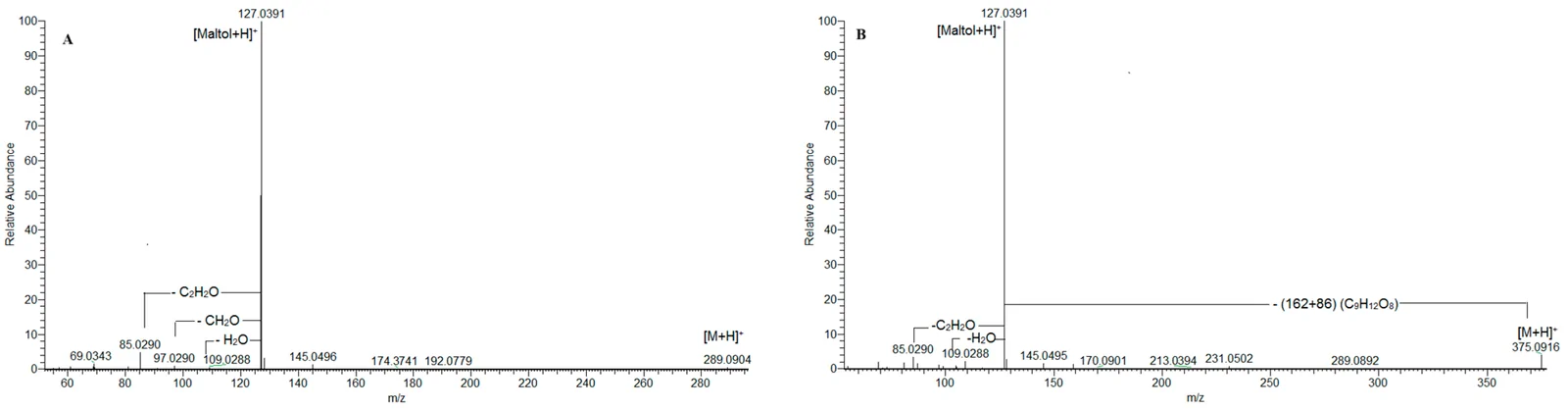

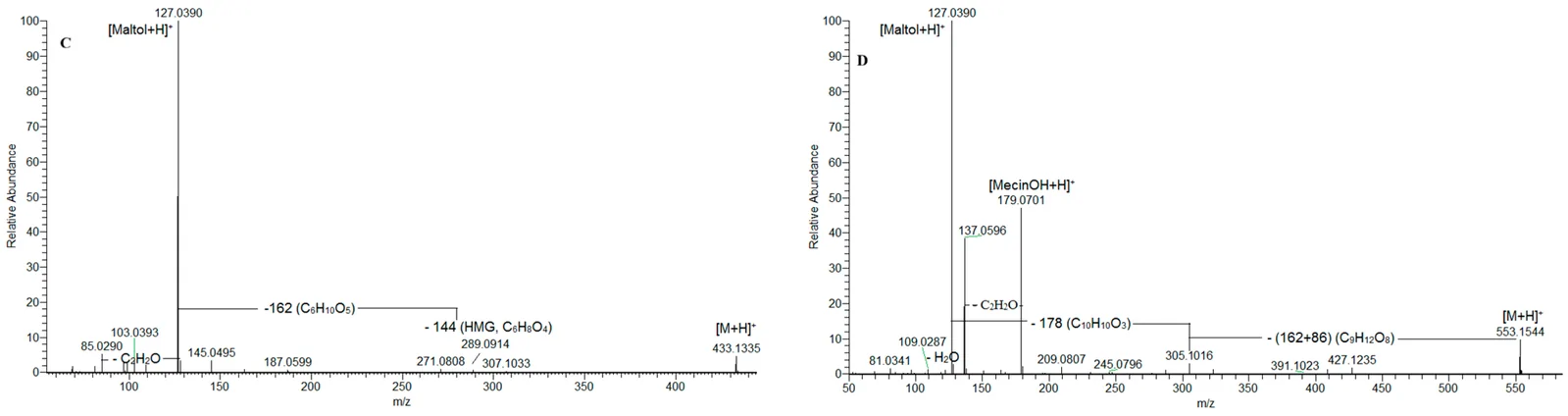
ESI-MS/MS spectra of maltol glycosides in positive ion mode: maltol hexoside (**64**) (**A**), maltol (malonyl)-hexoside (**69**) (**B**), maltol (hydroxy-methylglutaryl)-hexoside (**71**) (**C**), maltol [(malonylhexosyl)-methoxycinnamoyl]-hexoside (**74**) (**D**).

**Table 1:** Secondary metabolites in *Scleranthus perennis* methanol–aqueous extract in positive and negative ion mode.

№	Identified/Tentatively Annotated Compound	Molecular Formula	Exact Mass [M+H]^+^	Fragmentation Pattern in (+) ESI-MS/MS	Exact Mass [M−H]^−^	Fragmentation Pattern in (−) ESI-MS/MS	t_R_ (min) [M+H]^+^ [M−H]^−^	Δ ppm [M+H]^+^ [M−H]^−^	Confidence Level	Relative Content [%] *
**Flavonoids (*O*-, *C*- and *C*-, *O*-glycosides)**										
1	luteolin di-*C*-hexoside	C_27_H_30_O_16_	611.1607	611.1597 (100), 593.1494 (43.6), 575.1373 (11.8), 515.1176 (1.2), 491.1174 (9.6), 473.1070 (17.3), 443.0975 (13.5), 395.0757 (26.1), 365.0649 (20.6), 341.0653 (32.7), 311.0543 (29.78), 283.0592 (1.1), 195.0284 (3.8), 137.0231 (16.6), 135.0441 (6.0),	-	-	3.58	−1.622	D1	0.09
2	luteolin *C*-hexosyl-*C*-pentoside1	C_26_H_28_O_15_	581.1501	581.1484 (100), 563.1390 (24.3), 485.1076 (8.1), 395.0757 (24.0), 365.0653 (9.1), 341.0649 (15.3), 311.0545 (16.9), 137.0231 (8.3)	579.1355	579.1354 (100), 519.1127 (2.0), 489.1035 (14.3), 471.0918 (1.0), 453.0829 (0.9), 429.0828 (4.9), 411.0718 (2.8), 399.0717 (22.7), 381.0625 (1.8), 369.0612 (20.8), 339.0518 (2.0), 313.0716 (3.3), 312.0632 (3.5), 311.0567 (3.3), 298.0493 (3.1), 283.0619 (0.5), 133.0283 (4.5)	3.93	−2.919 −0.282	D1	0.82
3	luteolin *C*-hexosyl-*C*-pentoside 2	C_26_H_28_O_15_	581.1501	581.1496 (100), 563.1395 (27.1), 545.1289 (11.6), 527.1188 (7.7), 497.1078 (5.7), 443.0966 (11.4), 395.0757 (21.3), 365.0647 (13.8), 341.0651 (13.5), 311.0546 (22.3), 137.0234 (11.2)	579.1355	579.1355 (100), 489.1036 (6.6), 471.0930 (1.8), 459.0928 (9.8), 441.0821 (3.6), 429.0825 (6.8), 411.0723 (2.8), 399.0718 (21.6), 381.0617 (1.6), 369.0612 (17.22), 339.0500 (3.3), 313.0723 (3.7), 312.0631 (3.4), 311.0565 (3.7), 298.0490 (3.4), 283.0633 (0.6), 133.0281 (5.1)	4.10 4.12	−0.854 −0.075	D1	1.34
4	methylluteolin di-*C*-hexoside	C_28_H_32_O_16_	625.1763	625.1653 (100), 607.1653 (42.9), 589.1550 (5.9), 529.1342 (3.3), 505.1314 (7.0), 487.1226 (11.4), 457.1131 (10.0), 409.0916 (18.7), 379.0807 (15.8), 355.0807 (24.5), 337.0699 (14.8), 325.0702 (23.0), 310.0471 (4.7), 151.0389 (9.6), 149.0601 (3.3)	623.1618	623.1617 (100), 533.1321 (1.7), 503.1200 (10.9), 443.0981 (2.4), 413.0878 (13.9), 383.0772 (25.5), 365.0665 (0.2), 355.0844 (0.9), 323.0555 (1.5), 312.0638 (15.1), 311.0562 (7.3), 298.0489 (1.5)	4.19 4.18	−1.138 −0.125	D1	0.09
5	apigenin *C*-pentosyl-*C*-hexoside 1	C_26_H_28_O_14_	565.1552	565.1547 (100), 547.1445 (33.4), 529.1376 (5.0), 511.1234 (2.2), 499.1230 (8.7), 457.1092 (5.9), 433.0919 (13.6), 409.0917 (6.8), 379.0810 (26.7), 349.0708 (11.0), 325.0701 (17.7), 295.0601 (20.5), 121.0285 (12.1)	563.1406	563.1404 (100), 545.1284 (0.6), 527.1191 (0.5), 503.1168 (0.5), 473.1888 (9.6), 443.0984 (12.9), 413.0874 (3.1), 383.0770 (10.4), 353.0667 (14.8), 325.0718 (1.3), 297.0772 (7.4), 117.0331 (2.9)	4.32 4.34	−0.817 −0.122	D1	0.26
6	homoorientin ^a^	C_21_H_20_O_11_	-	-	447.0933	447.0932 (100), 429.0814 (3.6), 357.0616 (35.7), 327.0511 (51.7), 299.0551 (10.1), 285.0406 (5.5), 133.0281 (12.4)	4.55	−0.189	B	0.02
7	methylluteolin *C*-hexosyl-*C*-pentoside 1	C_27_H_30_O_15_	595.1657	595.1653 (100), 577.1547 (28.6), 559.1447 (6.4), 541.1357 (3.4), 529.1355 (8.7), 523.1231 (2.8), 505.1126 (3.3), 499.1230 (10.0), 481.1127 (12.3), 463.1028 (13.1), 409.0908 (33.64), 379.0802 (13.2), 355.0812 (18.7), 337.0695 (13.6), 325.0702 (18.6), 310.0475 (4.5), 195.0276 (1.0), 151.0391 (11.0), 149.0596 (4.4)	593.1512	593.1512 (100), 533.1346 (0.4), 503.1210 (7.1), 473.1084 (7.4), 413.0872 (9.8), 383.0771 (15.1), 353.0675 (0.3), 323.0577 (1.8), 312.0638 (14.0), 311.0568 (5.5), 298.0475 (2.1), 133.0285 (0.5)	4.56 4.59	−0.750 0.096	D1	1.3
8	apigenin *C*-pentosyl-*C*-hexoside 2	C_26_H_28_O_14_	565.1552	565.1548 (100), 547.1447 (24.7), 529.1334 (10.0), 511.1236 (7.8), 499.1224 (4.7), 481.1144 (7.7), 463.1021 (5.1), 409.0911 (16.8), 391.0807 (20.8), 379.0805 (15.0), 349.0702 (13.0), 325.0702 (11.9), 295.0598 (16.4), 121.0284 (9.9)	563.1406	563.1402 (100), 503.1196 (3.2), 473.1079 (5.4), 443.0978 (7.0), 383.0770 (17.3), 353.0667 (18.5), 297.0767 (9.9), 295.0621 (2.9), 253.0866 (0.8), 117.0331 (3.9)	4.56 4.57	−0.693 −0.761	D1	0.28
9	methylluteolin *C*-hexosyl-*C*-pentoside 2	C_27_H_30_O_15_	595.1657	595.1653 (100), 577.1547 (25.6), 559.1445 (9.7), 541.1339 (7.8), 529.1330 (4.2), 511.1239 (8.7), 493.1127 (6.1), 475.1013 (5.1), 463.1013 (9.7), 457.1131 (12.2), 439.1017 (19.1). 421.0913 (21.6), 409.0913 (19.8), 379.0807 (16.5), 355.0805 (13.1), 325.0705 (18.4), 310.0464 (4.2), 195.0294 (2.6), 177.0184 (1.1), 151.0391 (11.2), 149.0596 (4.0)	593.1512	593.1513 (100), 533.1309 (1.6), 503.1196 (3.9), 473.1088(8.9), 413.0875 (18.5), 383.0771 (23.6), 323.0558 (1.5), 312.0637 (15.0), 133.0283 (1.3)	4.63 4.67	−2.526 0.197	D1	2.06
10	methylluteolin *C*-hexosyl-*C*-pentoside 3	C_27_H_30_O_15_	595.1657	595.1654 (100), 577.1548 (61.7), 559.1450 (10), 541.1351(5.7), 529.1318 (7.5), 511.1205 (3.0), 499.1249 (7.3475.1194 (11.4), 463.1017 (10.8), 457.1143 (5.3), 439.1021 (11.1), 421.0915 (9.9), 409.0915 (36.3), 379.0811 (14.8), 355.0807 (18.3), 337.0702 (15.1), 325.0705 (21.1), 310.0473 (5.6), 195.0289 (1.3), 177.0188 (1.0), 151.0389 (10.1), 149.0598 (4.7)	593.1512	593.1511 (100), 533.1288 (0.5), 503.1194 (8.2), 473.1085 (10.0), 413.0880 (11.5), 383.0771 (16.7), 323.0559 (1.8), 312.0638 (13.1), 298.0482 (1.6), 133.0281 (0.6)	4.77 4.78	−1.487 −0.225	D1	0.68
11	apigenin *C-, O-*dihexoside	C_27_H_30_O_15_	595.1657	595.1653 (100), 577.1536 (7.3), 475.1192 (0.9), 433.1126 (97.9), 415.1022 (14.2), 397.0913 (21.4), 379.0807 (19.4), 337.0704 (45.8), 283.0597 (89.2), 267.0650 (13.2), 239.0700 (3.0), 255.0659 (0.9), 195.0291 (3.7), 121.0284 (7.5),	593.1512	593.1512 (100), 473.1093 (2.0), 413.0873 (13.0), 383.0775 (1.9), 353.0677 (1.4), 311.0551 (3.6), 293.0457 (32.6), 117.0022 (2.8)	4.84 4.85	−2.526 0.096	D1	0.11
12	methylluteolin (2″-*O*-hexosyl)-*C*-hexoside	C_28_H_32_O_16_	625.1763	625.1753 (100), 463.1228 (85.6), 445.1123 (9.0), 397.0918 (12.1), 367.0809 (37.3), 343.0808 (71.6), 313.0702 (76.2), 298.0468 (15.9), 151.0389 (4.6), 149.0594 (2.1)	623.1618	623.1614 (100), 443.0980 (12.6), 323.0560 (26.2), 308.0327 (8.5), 298.0480 (2.3)	5.09 5.08	−1.617 −0.510	D1	0.87
13	rutin ^a^	C_27_H_30_O_16_	611.1607	611.1607 (2.1), 465.1022 (14.7), 303.0496 (100), 257.0437 (1.0), 229.0504 (3.4), 153.0181 (3.1), 165.0182 (2.2), 137.0234 (2.5)	609.1461	609.1458 (100), 301.0349 (34.2), 300.0275 (58.4), 271.0249 (28.8), 255.0296 (12.6), 227.0343 (2.1), 178.9980 (2.7), 151.0023 (6.0), 107.0125 (2.5)	5.10 5.09	0.080 −0.489	B	0.05
14	kaempferol 3-*O*-deoxyhexosylhexoside	C_27_H_30_O_15_	-	-	593.1512	593.1512 (100), 473.1069 (0.6), 285.0387 (14.9), 284.0326 (86.2), 255.0326 (45.8), 227.0345 (28.8), 211.0394 (1.5), 178.99975 (0.9), 151.0023 (2.0), 107.0119 (0.9)	5.27	0.096	D1	0.08
15	apigenin *C*-pentosyl-*C*-hexoside 3	C_26_H_28_O_14_	565.1552	565.1545 (100), 547.1441 (33.1), 529.1331 (14.3), 511.1230 (8.9), 499.1235 (7.0), 451.1032 (3.0), 439.1017 (14.0), 409.0911 (17.8), 391.0800 (5.2), 355.0810 (7.9), 325.0701 (9.5), 337.0697 (8.2), 298.0824 (0.7), 121.0284 (0.9)	563.1406	563.1402 (100), 503.1199 (5.5), 473.1086 (13.0), 443.0970 (2.2), 413.0876 (13.9), 383.0773 (15.4), 353.0681 (0.4), 312.0639 (11.3), 311.0561 (6.4), 283.0612 (1.6)	5.29 5.29	−0.761	D1	1.35
16	hyperoside ^a^	C_21_H_20_O_12_	-	-	463.0885	463.0879 (100), 301.0348 (43.0), 300.0276 (70.6), 271.0248 (33.7), 255.0298 (12.4), 227.0337 (1.5), 178.9977 (3.2), 151.0024 (7.5)107.0122 (2.7)	5.30	−0.732	B	0.02
17	luteolin 7-*O*-glucoside ^a^	C_21_H_20_O_11_	449.1078	449.1071 (18.8), 287.0546 (100), 241.0490 (0.5), 153.0181 (5.7), 135.0442 (2.3), 137.0442 (0.5)	447.0933	447.0930 (100), 285.0402 (82.8), 256.0376 (3.2), 227.0351 (1.5), 175.0389 (1.4), 151.0023 (4.2), 133.0280 (3.8), 107.0125 (2.6)	5.39 5.40	−1.643 −0.295	B	0.51
18	methylluteolin *C*-hexoside	C_22_H_22_O_11_	463.1235	463.1233 (100), 445.1122 (28.4), 427.1025 (10.8), 409.0909 (14.6), 397.0915 (12.7), 379.0804 (9.1), 367.0809 (25.3), 343.0809 (44.3), 298.0471 (15.2)	461.1089	461.1093 (100), 371.0775 (23.9), 341.0664 (73.0), 313.0713 (4.2), 298.0481 (39.6)	5.43 5.44	−0.427 0.814	D1	0.06
19	luteolin (2″-*O*-hexosyl)-*C*-pentoside	C_26_H_28_O_15_	581.1501	581.1495 (100), 419.0967 (41.1), 401.0862 (19.8), 383.0756 936.5), 359.0758 (4.3), 329.0651 (40.5), 299.0546 (25.5), 287.0546 (28.8), 195.0287 (1.6), 137.0233 (7.7)	579.1355	579.1353 (100), 489.1040 (3.4), 399.0719 (28.1), 381.0634 (0.6), 357.0614 (15.8), 339.0499 (0.5), 327.0511 (48.7), 309.0406 (26.0), 299.0557 (4.1), 298.0480 (3.5), 297.0405 (3.8), 285.0405 (1.9), 175.0027 (4.2), 133.0281 (9.1)	5.54 5.55	−0.958 −0.403	D1	0.16
20	kaempferol 3-*O*-rutinoside ^a^	C_27_H_30_O_15_	595.1657	595.1627 (2.4), 449.1078 (12.8), 287.0546 (100), 269.0435 (0.3), 258.0519 (0.4), 241.0507 (0.3), 165.0183 (1.7), 121.0286 (1.9), 153.0181 (3.0)	593.1512	593.1511 (100), 285.0403 (63.6), 255.0297 (26.2), 227.0345 (18.2), 178.9969 (0.3), 151.0021 (1.3), 135.0070 (1.0), 107.0120 (1.5),	5.64 5.65	−6.928 −0.124	B	0.14
21	isorhamnetin 3-*O*-rutinoside ^a^	C_28_H_32_O_16_	-	-	623.1618	623.1616 (100), 315.0510 (79.1), 300.0273 (12.8), 271.0248 (24.5), 243.0295 (12.7), 227.0345 (0.8), 199.0390 (2.7), 151.0027 (3.9)	5.80	−0.318	B	0.03
22	methylluteolin (2″-*O*-acetylhexosyl)-*C*-pentoside	C_30_H_34_O_17_	667.1869	667.1863 (100), 463.1223 (16.2), 445.1121 (20.4), 427.1019 (11.5), 409.0939 (3.8), 367.0805 (10.2), 355.0790 (0.8), 343.0807 (95.4), 328.0576 (15.9), 315.0843 (0.8), 313.0707 (14.9), 151.0388 (6.8)	665.1723	665.1726 (100), 443.0974 (26.5), 323.0560 (63.0), 308.0326 (23.2), 280.0376 (15.3),	5.83 5.84	−0.893 0.432	D1	0.05
23	apigenin (2″-*O*-hexosyl)-*C*-pentoside	C_26_H_28_O_14_	565.1552	565.1547 (100), 403.1018 (41.1), 385.0912 (19.9), 367.0809 (35.2), 343.0810 (4.4), 337.0702 (12.8), 313.0701 (35.2), 295.0601 (9.6), 284.0601 (17.3), 283.0597 (24.7), 271.0598 (27.1), 256.0735 (2.9), 255.0649 (2.0), 195.0292 (1.5), 177.0177 (0.3), 121.0287 (7.8), 119.0492 (2.0)	563.1406	563.1403 (100), 383.0770 (54.8), 293.0456 (53.9), 249.0551 (0.6), 117.0332 (9.7)	5.87 5.89	−0.923 −0.548	D1	0.25
24	acetylluteolin (2″-*O*-hexosyl)-*C*-pentoside 1	C_28_H_30_O_16_	623.1607	623.1597 (100), 461.1070 (36.2), 443.0967 (8.6), 401.0861 (9.4), 383.0753 (26.5), 365.0652 (7.7), 353.0646 (3.3), 341.0654 (4.4), 327.0867 (2.4), 323.0548 (2.7), 313.0702 (8.0), 299.0547 (43.0), 287.0545 (19.2), 249.0392 (0.5), 229.0494 (1.5)153.0178 (0.5), 137.0233 (4.6)	621.1461	621.1460 (100), 579.1347 (0.3), 561.1214 (5.3), 441.0826 (9.2), 399.0714 (10.3), 381.0619 (9.0), 327.0511 (48.0), 309.0511 (15.2), 298.0484 (5.3), 285.0402 (1.1), 267.0299 (1.1), 175.0030 (2.5), 133.0281 (11.4)	5.97 5.97	−3.254 −0.174	D1	0.40
25	apigenin 7-*O*-glucoside ^a^	C_21_H_20_O_10_	433.1129	433.1124 (16.3), 271.0597 (100), 243.0639 (0.4), 225.0537 (0.3), 163.0383 (0.2), 153.0182 (5.0), 119.0494 (1.9), 121.0286 (0.4)	431.0984	431.0981 (100), 269.0447 (24.2), 268.0377 (54.3), 211.0387 (1.1), 151.0026 (2.7), 117.0330 (1.4), 107.0125 (2.0)	6.08 6.10	−0.493 −0.260	B	0.06
26	acetylapigenin (2″-*O*-hexosyl)-*C*-pentoside	C_28_H_30_O_15_	607.1657	607.1647 (100), 445.1122 (51.1), 427.1014 (10.0), 409.0914 (9.5), 385.0915 (13.1), 367.0807 (37.8), 349.0698 (8.4), 325.0704 (6.5), 311.0913 (4.2), 307.0598 (2.5), 283.0596 (53.6), 271.057 (27.7), 163.0385 (0.5), 153.0179 (0.6), 119.0490 (1.6), 121.0285 (5.8)	605.1512	605.1514 (100), 563.1447 (2.8), 545.1297 (0.5), 425.0878 (40.4), 383.0772 (17.0), 365.0670 (7.1), 335.0559 (4.5), 293.04257 (34.7), 269.0453 (2.5), 249.0533 (0.6), 175.0028 (4.1), 117.0331 (10.0)	6.28 6.30	1.674 0.292	D1	0.71
27	methylluteolin *O*-hexoside	C_22_H_22_O_11_	463. 1235	463.1227 (28.5), 301.0702 (100), 286.0467 (36.5), 258.0519 (18.3), 153.0187 (0.3)	-	-	6.31	−4.048	D1	0.12
28	chrysoeriol (2″-*O*-hexosyl)-*C*-pentoside	C_27_H_30_O_15_	595.1657	595.1651 (100), 433.1121 (31.0), 415.1014 (17.2), 397.0809 (29.8), 367.0803 (10.4), 343.0807 (31.1), 325.0702 (6.9), 313.0701 (18.3), 301.0703 (19.8), 195.0293 (0.7)	593.1512	593.1512 (100), 413.0877 (46.1), 323.0562 (27.5), 308.0326 (18.0), 280.0377 (10.7)	6.41 6.42	−2.929 −0.22	D1	0.90
29	apigenin (2″-*O*-acetylhexosyl)-*C*-pentoside	C_28_H_30_O_15_	607.1657	607.1649 (100), 403.1018 (34.2), 385.0913 (22.2), 367.0807 (27.8), 343.0804 (3.8), 313.0703 (64.0), 295.0597 (8.5), 283.0597 (20.1), 271.0596 (14.4), 243.0653 (1.1), 153.0174 (0.6), 121.0286 (8.6), 119.0493 (1.9)	605.1512	605.1514 (6.5), 383.0772 (57.2), 293.0456 (62.5), 265.0515 (1.1), 249.0559 (1.0), 237.0534 (0.4), 117.0331 (10.3),	6.47 6.49	1.460 0.207	D1	0.45
30	isovitexin ^a^	C_21_H_20_O_10_	-	-	431.0984	431.0981 (100), 413.0893 (3.9), 371.0772 (28.3), 341.0664 (48.1), 298.0482 (28.2)	6.50	−0.290	B	0.02
31	acetylluteolin (2″-*O*-hexosyl)-*C*-pentoside 2	C_28_H_30_O_16_	623.1607	623.1599 (100), 461.1070 (41.7), 443.0966 (12.8), 383.0753 (48.6), 365.0649 (15.0), 329.0651 (59.5), 299.0546 (43.1), 287.0545 (19.7), 229.0491 (2.2), 153.0188 (0.7), 137.0233 (11.4), 135.0441 (2.8)	621.1461	621.1459 (100), 579.1348 (3.6), 441.0826 (12.2), 381.0613 (10.3), 339.0506 (7.8), 327.0510 (60.6), 309.046 (15.8), 298.0483 (6.0), 285.0402 (0.7), 133.0282 (10.5)	6.74 6.74	−1.205 −2.965	D1	1.03
32	vitexin ^a^	C_21_H_20_O_10_	433.1129	433.1124 (100), 415.1019 (11.7), 397.0913 (35.0), 373.0912 (8.9), 343.0807 (18.2), 313.0699 (12.1), 285.0764 (1.2), 284.0670 (1.8), 283.0595 (0.5), 271.0590 (1.3), 243.0644 (1.6), 153.0168 (0.3)	431.0984	431.0981 (100), 371.0772 (6.6), 341.0665 (62.8), 298.0482 (30.0), 269.0456 (2.6), 227.0352 (0.7)	6.76 6.77	−1.208 −0.626	B	0.08
33	acetylapigenin (2″-*O*-acetylhexosyl)-*C*-pentoside	C_30_H_32_O_16_	649.1763	649.1754 (100), 445.1123 (56.5), 427.1021 (12.5), 409.0913 (12.0), 385.0909 (14.5), 367.0808 (34.5), 349.0703 (6.7), 325.0703 (6.3), 311.0910 (3.2), 283.0596 (53.9), 271.0595 (14.4), 153.0184 (0.6), 121.0390 (7.4), 19.0490 (2.4)	647.1618	647.1620 (100), 425.0878 (48.1), 383.0772 (15.8), 365.0654 (6.4), 335.0562 (5.7), 311.0566 (0.4), 293.0457 (36.7), 269.0455 (2.8), 265.0498 (0.5), 237.0551 (0.4), 117.0331 (10.5)	6.80 6.81	−0.911 0.359	D1	1.19
34	acetyl-methylluteolin (2″-*O*-hexosyl)-*C*-pentoside 1	C_29_H_32_O_16_	637.1763	637.1753 (100), 475.1231 (51.6), 457.1128 (14.1), 439.1019 (13.1), 415.1020 (21.0), 397.0913 (47.5), 379.0804 (12.2), 369.0542 (4.4), 355.0805 (7.4), 341.0648 (6.6), 313.0701 (72.5), 301.0703 (31.2), 298.0470 (22.8), 151.0390 (9.4), 149.0592 (2.1)	635.1618	635.1616 (100), 593.1519 (3.7), 455.0981 (26.2), 440.0743 (7.3), 413.0875 (16.1), 395.0773 (4.5), 365.0674 (2.0), 323.0560 (17.6), 308.0327 (6.9), 280.0378 (3.7)	6.82 6.82	−1.603 −0.249	D1	2.51
35	methylluteolin (2″-*O*-acetylhexosyl)-*C*-pentoside	C_29_H_32_O_16_	637.1763	637.1755 (100), 547.1445 (0.5), 433.1125 (24.8), 415.1017 (19.3), 397.0914 (23.4), 367.0804 (8.3), 343.0808 (49.8), 328.0575 (7.5), 315.0827 (1.0), 313.0699 (13.0), 301.0701 (10.0), 195.0288 (2.0), 151.0389 (4.9)	635.1618	635.1616 (100), 413.0876 (45.0), 398.0639 (5.3), 353.0679 (1.0), 323.0560 (28.9), 308.0328 (19.6), 280.0377 (13.2), 133.0275 (0.2)	7.04 7.05	−1.304 −0.217	D1	1.32
36	acetylapigenin (2″-*O*-hexosyl)-*C*-pentoside	C_28_H_30_O_15_	607.1657	607.1650 (100), 445.1125 (47.1), 427.1021 (11.9), 409.0918 (2.6), 385.0913 (3.5), 367.0808 (42.2), 349.0706 (12.8), 313.0702 (52.9), 295.0598 (15.9), 283.0598 (41.4), 271.0599 (17.1), 163.0385 (0.5), 121.0286 (13.8), 119.0494 (2.5)	605.1512	605.1514 (100), 563.1420 (1.3), 545.1300 (0.3), 425.0880 (33.9), 383.0772 (7.9), 365.0661 (3.8), 335.0555 (2.0), 311.0560 (10.7), 293.0456 (52.1), 269.0452 (1.0), 237.0550 (0.5), 117.0331 (12.1)	7.17 7.18	−0.747 0.292	D1	1.28
37	apigenin [2″-*O*-(diacetylhexosyl)]-*C*-pentoside	C_30_H_32_O_16_	649.1763	649.1755 (100), 403.1018 (31.3), 385.0913 (21.7), 367.0808 (27.2), 343.0806 (2.9), 313.0701 (55.7), 283.0598 (20.5), 271.0597 (12.2), 13.0179 (0.7), 121.0285 (8.6), 119.0495 (1.8)	647.1618	647.1617 (100), 383.0772 (60.1), 293.0456 (72.5), 269.0457 (1.1), 221.0599 (0.6), 117.0331 (11.6)	7.19 7.21	−0.811 −0.028	D1	0.17
38	methylluteolin [2″-*O*-(diacetylhexosyl)]-*C*-pentoside 1	C_31_H_34_O_17_	679.1869	679.1861 (100), 475.1231 (48.6), 457.1125 (12.4), 439.1023 (9.3), 415.1016 (16.8), 397.0911 (32.3), 379.0807 (6.5), 355.0803 (4.0), 341.0648 (4.9), 313.0703 (49.1), 301.0704 (10.4), 298.0474 (16.6), 286.0482 (4.4), 270.0523 (1.9), 151.0391 (5.1), 149.0580 (1.1),	677.1723	677.1722 (100), 635.1619 (4.1), 455.0981 (23.3), 440.0755 (9.4), 413.0867 (15.7), 395.0790 (4.1), 365.0668 (2.5), 341.0666 (5.6), 323.0557 (19.4), 308.0327 (10.5), 280.0378 (4.4),	7.38 7.38	−1.142 −0.211	D1	5.40
39	acetyl-methylluteolin (2″-*O*-hexosyl)-*C*-pentoside 2	C_29_H_32_O_16_	637.1763	637.1755 (100), 475.1227 (28.5), 457.1117 (9.3), 415.1031 (3.1), 397.0913 (31.9), 379.0805 (7.5), 369.0971 (1.8), 343.0808 (38.2), 355.0826 (0.6), 313.0699 (20.1), 301.0699 (12.1), 286.0470 (3.1), 272.0683 (2.1), 258.0514 (0.6), 195.0287 (1.9), 151.0389 (5.8), 149.0599 (0.8)	635.1618	635.1617 (100), 593.1495 (2.1), 455.0980 (24.2), 440.0749 (3.8), 413.0875 (7.7), 395.0768 (2.3), 365.0662 (0.8), 323.0563 (22.7), 308.0328 (12.0), 280.0375 (9.1)	7.51 7.52	−1.304 −0.123	D1	7.93
40	acetylapigenin [2″-*O*-(diacetylhexosyl)]-*C*-pentoside 1	C_32_H_34_O_17_	691.1869	691.1858 (100), 445.1125 (52.9), 427.1020 (12.0), 409.0908 (12.6), 385.0909 (16.2), 367.0807 (37.1), 325.0706 (7.6), 283.0597 (52.5), 271.0598 (14.9), 121.0286 (7.2), 119.0494 (1.7)	689.1723	689.1721 (100), 425.0876 (55.8), 383.0769 (13.4), 335.0552 (6.9), 311.0559 (3.1), 293.0456 (38.3), 117.0331 (10.9)	7.55 7.56	−1.571 −0.381	D1	1.76
41	methylluteolin [2″-*O*-(diacetylhexosyl)]-C-pentoside 2	C_31_H_34_O_17_	679.1869	679.1858 (100), 475.1260 (0.6), 433.1123 (26.5), 415.1016 (19.4), 397.0913(25.4), 367.0806 (9.4), 328.0576 (8.9), 313.0703 (15.3), 301.0702 (11.4), 286.0473 (3.1), 151.0391 (4.7), 149.0594 (1.2),	-	-	7.75		D1	0.35
42	acetylapigenin (2″-*O*-acetylhexosyl)-*C*-pentoside	C_30_H_32_O_16_	649.1763	649.1757 (100), 445.1124 (29.9), 427.1016 (14.9), 367.0809 (41.3), 349.0707 (14.5), 313.0703 (96.7), 283.0597 (31.4), 121.0286 (11.0), 119.0492 (2.6)	647.1618	647.1619 (100), 605.1535 (0.6), 515.1177 (1.9), 425.0878 (42.6), 383.0772 (9.3), 365.0664 (5.6), 335.0574 (2.5), 311.0562 (14.8), 293.0457 (73.3), 269.0460 (1.9), 163.0384 (1.7), 117.0331 (13.6)	7.85 7.87	−0.911 0.266	D1	6.56
43	methylluteolin [2″-*O*-(diacetylhexosyl)]-*C*-pentoside 3	C_31_H_34_O_17_	-	-	677.1723	677.1721(100), 455.0983 (26.1), 440.0751 (4.8), 413.0871 (7.8), 395.0769 (2.9), 365.0679 (0.8), 341.0666 (8.3), 323.0560 (28.9), 308.0327 (15.6), 280.0375 (9.6)	7.94	−1.806	D1	1.25
44	acetylapigenin [2″-*O*-(methoxycinnamoylhexosyl)]-*C*-pentoside 1	C_38_H_40_O_18_	785.2287	785.2278 (28.0), 403.1016 (3.6), 367.0806 (3.2), 343.0810 (0.4), 313.0702 (5.3), 295.0590 (1.7), 283.0594 (2.6), 271.0595 (3.5), 179.0702 (56.0), 151.0751 (1.2), 137.0597 (100), 121.0285 (1.7), 119.0491 (1.0),	783.2142	783.2145 (95.9), 605.1530 (2.2), 561.1411 (2.0), 383.0715 (80.0), 293.0456 (100.0), 269.0458 (1.6), 175.0027 (12.4), 117.0331 (16.6)	8.06 8.08	−0.961 0.353	D1	0.21
45	acetyl-methylluteolin [2″-*O*-(diacetylhexosyl)]-*C*-pentoside 1	C_33_H_36_O_18_	721.1974	721.1965 (100), 475.1229 (48.2), 457.1125 (15.4), 439.1019 (14.2), 415.1017 (18.1), 397.0913 (39.8), 379.0809 (8.3), 367.0789 (0.6), 355.0801 (5.1), 313.0701 (46.6), 298.0467 (16.1), 286.0473 (5.0), 151.0390 (5.4), 149.0596 (1.5)	719.1829	719.1830 (100), 455.0982 (37.7), 413.0873 (14.8), 341.0671 (2.3), 323.0558 (22.4), 308.0326 (9.9), 280.0378 (5.5)	8.13 8.16	−2.867 0.153	D1	2.78
46	acetylapigenin [2″-*O*-(methoxycinnamoylhexosyl)]-*C*-pentoside 2	C_38_H_40_O_18_	785.2287	785.2278 (32.2), 445.1123 (9.7), 409.0901 (2.0), 367.0812 (6.2), 341.1229 (6.7), 325.0717 (1.3), 283.0597 (8.7), 271.0598 (5.0), 179.0701 (57.8), 164.0465 (1.7), 137.0597 (100), 119.0493 (1.0)	783.2142	783.2145 (100), 725.0877 (62.9), 383.0770 (8.0), 365.0670 (7.4), 335.0572 (9.0), 293.0456 (28.2), 269.0454 (1.7), 237.0760 (4.2), 175.0023 (3.5), 117.0332 (10.0)	8.17 8.19	0.353 0.450	D1	0.09
47	acetylapigenin [2″-*O*-(galloylhexosyl)]-*C*-pentoside 1	C_35_H_36_O_20_	777.1873	777.1857 (100), 445.1123 (47.7), 427.1013 (15.2), 409.0911 (13.0), 385.0911 (17.7), 367.0807 (39.4), 349.0701 (10.8), 325.0702 (6.9), 283.0597 (50.7), 271.0597 (22.0), 127.0390 (29.5), 109.0287 (58.0), 121.0286 (0.4), 119.0493 (2.5)	-	-	8.19	−1.540	D1	1.57
48	acetyl-methylluteolin (2″-*O*-acetylhexosyl)-*C*-pentoside	C_31_H_34_O_17_	679.1869	679.1862 (100), 475.1227 (25.3), 457.1116 (15.0), 415.1012 (4.0), 397.0913 (40.9), 379.0801 (11.0), 343.0807 (92.7), 313.0703 (29.5), 298.0471 (10.4), 286.0474 (2.9), 301.0703 (8.5), 195.0289 (4.7), 151.0390 (9.8), 149.0594 (1.8),	677.1723	677.1719 (100), 635.1639 (3.3), 455.0980 (38.0), 440.0748 (8.2), 413.0886 (11.5), 395.0778 (4.2), 365.0643 (1.3), 341.0665 (15.9), 323.0560 (44.1), 308.0326 (16.5), 280.0375 (10.8)	8.26 8.26	−0.995 −0.654	D1	15.29
49	acetyl-methylluteolin [2″-*O*-(triacetyl-hexosyl)]-*C*-pentoside 1	C_35_H_36_O_19_	-	-	761.1935	761.1936 (100), 497.1105 (3.6), 455.0985 (44.5), 440.0752 (13.2), 413.0865 (5.3), 365.0656 (4.4), 323.0561 (16.7), 308.0328 (7.0), 133.0284 (0.9)	8.54	0.148	D1	0.19
50	acetylapigenin [2″-*O*-(diacetylhexosyl)]-*C*-pentoside 2	C_32_H_34_O_17_	691.1869	691.1855 (100), 445.1122 (35.6), 427.1021 (13.6), 409.0893 (3.2), 385.0916 (4.4), 367.0807 (46.2), 313.0701 (95.7), 283.0598 (36.5), 271.0598 (10.1), 121.0285 (12.6), 119.0491 (2.5)	689.1723	689.1721 (100), 647.1656 (1.6), 425.0877 (42.4), 383.0760 (7.4), 335.0562 (2.8), 311.0562 (5.7), 293.0456 (73.8), 269.0471 (0.6), 249.0542 (0.5), 237.0560 (0.4), 117.0331 (14.4)	8.63 8.65	−2.005 −0.294	D1	8.28
51	acetylapigenin [2″-*O*-(methoxycinnamoylhexosyl)]-*C*-pentoside 2	C_38_H_40_O_18_	785.2287	785.2217 (27.4), 445.1112 (11.4), 427.1003 (1.7), 409.0919 (0.2), 385.0916 (0.8), 367.0806 (6.5), 349.0703 (2.2), 341.1226 (15.7), 313.0700 (8.9), 295.0599 (2.6), 283.0597 (6.3), 271.0596 (3.4), 179.0701 (67.3), 164.0464 (2.0), 151.0754 (1.3), 137.0596 (100), 121.0285 (1.7), 119.0491 (1.0),	783.2142	783.2144 (100), 425.0879 (38.0), 383.0754 (6.5), 365.0678 (4.4), 335.0572 (1.3), 293.0458 (71.0), 249.0565 (1.0), 237.0770 (3.8), 175.0028 (10.1), 117.0332 (15.0)	8.65 8.66	−1.081 0.233	D1	0.14
52	acetyl-methylluteolin [2″-*O*-(triacetyl-hexosyl)]-*C*-pentoside 1	C_35_H_36_O_19_	-	-	761.1935	761.1935 (100), 719.1808 (6.3), 455.0984 (41.4), 440.0747 (11.5), 413.0878 (21.7), 365.0662 (4.6), 323.0560 (36.07), 308.0328 (16.1), 280.0376 (8.5), 133.0275 (0.7)	8.82	0.088	D1	0.65
53	luteolin ^a^	C_15_H_10_O_6_	-	-	285.0405	285.0403 (100), 229.0495 (0.6), 211.0392 (0.6), 151.0020 (0.7), 107.0123 (1.0)	8.86	−0.425	B	0.03
54	acetylapigenin [2″-*O*-(galloylhexosyl)]-*C*-pentoside 2	C_35_H_36_O_20_	777.1873	777.1865 (100), 445.1127 (27.5), 427.1021 (10.1), 409.0905 (1.9), 385.0921 (3.4), 367.0808 (28.0), 349.0701 (11.3), 313.0703 (73.7), 295.0600 (13.0), 283.0599 (26.6), 271.0589 (5.6), 243.0653 (1.7), 171.0288 (0.8), 127.0391 (79.5), 109.0288 (62.4), 121.0286 (9.5), 119.0494 (2.0)	-	-	8.96	−0.750	D1	0.99
55	acetyl-methylluteolin [2″-*O*-(diacetyl-hexosyl)]-*C*-pentoside 2	C_33_H_36_O_18_	721.1974	721.1964 (89.4), 475.1230 (28.7), 457.1122 (15.7), 415.1035 (4.3), 397.0913 (45.1), 379.0809 (13.3), 343.0807 (100), 328.0573 (18.2), 313.0704 (35.3), 151.0340 (9.7), 149.0593 (2.1)	719.1829	719.1837 (100), 455.0997 (28.8), 440.0753 (5.4), 413.0885 (5.3), 341.0652 (3.7), 323.0561 (27.0), 308.0332 (16.2), 280.0392 (9.5)	9.05 9.06	−1.031 1.158	D1	12.36
56	acetylapigenin [2″-*O*-(galloylhexosyl)]-*C*-pentoside 3	C_35_H_36_O_20_	777.1873	777.1860 (100), 445.1125 (21.8), 427.1022 (9.4), 409.0908 (2.1), 385.0905 (4.7), 367.0809 (30.7), 349.0708 (12.2), 313.0702 (99.0), 295.0598 (15.0), 283.0598 (32.1), 271.0598 (11.3), 243.0648 (1.8), 171.0271 (0.5), 127.0390 (23.2), 109.0287 (48.2), 121.0286 (14.7), 119.0495 (3.0)	--	-	9.15	−1.230	D1	7.17
57	acetyl-methylluteolin [2″-*O*-(triacetyl-hexosyl)]-*C*-pentoside 3	C_35_H_36_O_19_	-	-	761.1935	761.1935 (100), 719.1841 (6.0), 497.1085 (18.9), 455.0984 (19.6), 440.0755 (3.4), 413.0877 (2.7), 365.0682 (1.2), 323.0560 (18.1), 308.0322 (8.0), 280.0375 (4.9)	9.18	0.088	D1	0.93
58	acetyl-methylluteolin [2″-*O*-(methoxy-cinnamoylhexosyl)]-*C*-pentoside 1	C_41_H_42_O_20_	-	-	855.2353	855.2352 (100), 677.1741 (2.0), 455.0982 (31.8), 440.0749 (5.6), 413.0880 (3.6), 365.0666 (1.0), 323.0559 (34.9), 308.0325 (22.9), 280.0374 (13.9)	9.28	−0.101	D1	0.84
59	acetyl-methylluteolin [2″-*O*-(triacetyl-hexosyl)]-*C*-pentoside 4	C_35_H_36_O_19_	-	-	761.1935	761.1937 (100), 719.1838 (2.4), 455.0982 (49.3), 440.0743 (11.5), 413.0878 (6.2), 365.0674 (2.3), 323.0561 (55.0), 308.0326 (29.8), 280.0374 (17.6)	9.57	0.208	D1	2.02
60	acetyl-methylluteolin [2″-*O*-(methoxy-cinnamoylhexosyl)]-*C*-pentoside 2	C_41_H_42_O_20_	-	-	855.2353	855.2349 (100), 677.1655 (1.0), 455.0981 (49.2), 440.0733 (8.4), 413.0887 (5.9), 365.0697 (1.2), 323.0561 (53.6), 308.0324 (24.7), 280.0379 (19.1)	9.69	−0.464	D1	1.978
61	acetyl-methylluteolin [2″-*O*-(triacetyl-hexosyl)]-*C*-pentoside 5	C_35_H_38_O_19_	763.2080	763.2073 (100), 475.1229 (18.7), 457.1122 (9.6), 397.0914 (29.8), 343.0808 (85.6), 328.0808 (12.6), 313.0703 (24.7), 298.0473 (9.7)	761.1935	761.1938 (100), 719.1875 (2.5), 455.0983 (45.4), 440.0752 (9.8), 413.0885 (5.2), 365.0674 (1.1), 323.0561 (51.0), 308.0326 (25.5), 280.0376 (17.6)	10.11 10.13	−0.924 0.398	D1	0.43
62	acetyl-methylluteolin [2″-*O*-(methoxy-cinnamoylhexosyl)]-*C*-pentoside 3	C_41_H_42_O_20_	-	-	855.2353	855.2355 (100), 455.0984 (31.0), 440.0760 (6.2), 413.0870 (5.9), 365.0675 (1.5), 323.0561 (34.6), 308.0327 (18.2), 280.0375 (13.3)	10.18	0.214	D1	0.21
**Maltol-glycosides**										
63	maltol dihexoside 1	C_18_H_26_O_13_	451.1446	451.1423 (0.9), 289.0913 (16.8), 127.0390 (100), 109.0287 (0.7), 97.0288 (2.7), 85.0290 (7.5)	-	-	1.28	−2.287	D1	0.30
64	maltol hexoside 1	C_12_H_16_O_8_	289.0918	289.0904 (0.5), 127.0391 (100), 109.0288 (0.6), 97.0290 (1.0), 85.0290 (4.7)	-	-	1.52	−1.374	D1	10.94
65	maltol hexoside 2	C_12_H_16_O_8_	289.0918	289.0918 (0.5), 127.0391 (100), 109.0288 (0.5), 97.0290 (1.2), 85.0290 (4.5)	-	-	1.93	−0.024	D1	3.41
66	maltol (pentosyl)-hexoside	C_17_H_24_O_12_	421.1341	421.1333 (0.3), 289.0914 (15.0), 127.0391 (100), 109.0288 (1.7), 97.0288 (1.7), 85.0291 (3.2)	-	-	1.95	−0.723	D1	2.96
67	maltol dihexoside 2	C_18_H_26_O_13_	451.1446	451.1434 (1.1), 289.0912 (7.9), 127.0391 (100), 109.0287 (0.9), 97.0290 (2.1), 85.0290 (8.2)	-	-	1.98	−1.187	D1	1.58
68	maltol (malonyl)-hexoside 1	C_15_H_18_O_11_	375.0922	375.0905 (1.1), 127.0390 (100), 109.0285 (0.9), 97.0289 (1.0), 85.0290 (1.8)	-	-	2.87	−2.256	D1	0.72
69	maltol (malonyl)-hexoside 2	C_15_H_18_O_11_	375.0922	375.0916 (4.1), 289.0892 (0.1), 127.0391 (100), 109.0288 (1.2), 97.0288 (1.2), 85.0290 (3.4)	-	-	3.12	−0.578	D1	4.65
70	maltol (malonylhexosyl)-hexoside	C_21_H_28_O_16_	537.1450	537.1443 (7.8), 375.0916 (30.2), 127.0390 (100), 109.0287 (3.8), 97.0288 (3.1)	-	-	3.35	−0.421	D1	2.50
71	maltol (hydroxy-methylglutaryl)-hexoside	C_18_H_24_O_12_	433.1341	433.1335 (5.8), 289.0914 (0.9), 127.0391 (100), 109.0288 (2.2), 97.0289 (3.2), 85.0291 (4.4)	-	-	3.69	−0.603	D1	6.85
72	maltol (dimalonyl)-hexoside 1	C_18_H_20_O_14_	461.0926	461.0917 (7.3), 213.0394 (1.4), 127.0390 (100), 109.0287 (4.8), 97.0289 (6.2), 85.0290 (1.5)	-	-	3.84	−1.825	D1	0.12
73	maltol (dimalonyl)-hexoside 2	C_18_H_20_O_14_	461.0926	461.0943 (15.0), 213.0395 (1.5), 127.0390 (100), 109.0287 (7.7), 97.0288 (3.3), 85.0291 (1.1)	-	-	4.12	3.727	D1	0.21
74	maltol [(malonylhexosyl)-methoxycinnamoyl]-hexoside	C_31_H_38_O_19_	715.2080	715.2050 (0.4), 553.1543 (29.7), 305.1015 (3.3), 179.0701 (61.9), 137.0596 (41.5), 127.0390 (100), 109.0289 (1.9), 97.0288 (1.8), 85.0288 (0.5)	-	-	6.63	−4.174	D1	46.42
75	maltol [(malonyl)-methoxycinnamoyl]-hexoside 1	C_25_H_28_O_14_	553.1552	553.1544 (10.3), 305.1016 (3.1), 179.0701 (47.5), 137.0596 (39.2), 127.0390 (100), 109.0287 (1.4), 97.0289 (1.2), 85.0290 (0.4)	-	-	6.63	−1.377	D1	12.91
76	maltol [(malonyl)-methoxycinnamoyl]-hexoside 2	C_25_H_28_O_14_	553.1552	553.1550 (12.0), 305.1020 (3.1), 179.0702 (46.4), 164.0466 (1.3), 137.0597 (41.2), 127.0391 (100), 109.0286 (1.4), 97.0291 (1.5), 85.0290 (1.3)	-	-	7.08	−0.329	D1	6.51
**Phenolic acids, derivatives, and coumarins**										
77	hydroxybenzoic acid-*O*-hexoside 1	C_13_H_16_O_8_	-	-	299.0778	299.0774 (9.5), 137.0231 (100), 93.0330 (59.3)	1.30	0.533	D1	0.52
78	protocatechuic acid-*O*-hexoside	C_13_H_16_O_9_	-	-	315.0727	315.0722 (100), 153.0182 (25.6), 152.0103 (52.0), 123.0074 (3.6), 109.0288 (8.1), 108.0202 (89.5)	1.67	0.142	D1	0.70
79	vanillic acid-*O*-hexoside	C_14_H_18_O_9_	-	-	329.0875	329.0882 (2.5),167.0339 (100), 152.0102 (20.5), 123.0437 (0.2), 108.0202 (31.3)	1.80	1.199	D1	0.93
80	protocatechuic acid ^a^	C_7_H_6_O_4_	-	-	153.0182	153.0182 (18.0), 109.0280 (100), 91.0174 (1.9)	2.06	−7.397	B	0.44
81	syringic acid-*O*-hexoside, 1	C_15_H_20_O_10_	-	-	359.0985	359.0984 (10.1), 197.0447 (100), 182.0210 (19.4), 166.9976 (8.7), 153.0546 (14.5), 138.0309 (26.0), 123.0074 (30.3)	2.30	0.084	D1	0.25
82	caffeic acid-*O*-hexoside	C_15_H_18_O_9_	-	-	341.0871	341.0877 (31.4), 179.0340 (22.1), 161.0233 (100), 135.0438 (6.7), 133.0281 (27.0)	2.66	−0.367	D1	0.28
83	4-hydroxybenzoic acid ^a^	C_7_H_6_O_3_	-	-	137.0230	137.0231 (100), 119.0126 (0.9), 109.0277 (5.6), 108.0205 (10.1), 93.0330 (27.8), 65.0380 (1.4)	2.84	−9.614	B	15.09
84	ferulic acid *O*-hexoside 1	C_16_H_20_O_9_	-	-	355.1035	355.1022 (1.0), 193.0497 (100), 178.0261 (9.6), 149.0595 (22.3), 134.0360 (54.5)	2.98	−3.535	D1	1.27
85	3-hydroxybenzoic acid ^a^	C_7_H_6_O_3_	-	-	137.0230	137.0232 (15.7), 108.0204 (4.0), 93.0330 (100)	3.01	−8.884	B	14.70
86	hydroxybenzoic acid-*O*-hexoside 2	C_13_H_16_O_8_	-	-	299.0778	299.0765 (2.0), 137.0231 (100), 93.0330 (45.4)	2.47	−2.476	D1	0.02
87	dihydrocaffeic acid O-hexoside	C_15_H_20_O_9_	-	-	343.1035	343.1034 (30.4), 181.0497 (100), 137.0595 (18.2), 121.0282 (6.0), 109.0280 (1.9)	3.19	−0.161	D1	0.12
88	quinic acid	C_7_H_12_O_6_	-	-	191.0549	191.0552 (100), 173.0447 (0.9), 127.0385 (3.1), 111.0435 (1.3), 93.0330 (6.2), 85.0279 (32.1)	3.19	−4.561	D1	0.18
89	chlorogenic acid ^a^	C_16_H_18_O_9_	-	-	353.0878	353.0878 (5.7), 191.0552 (100), 179.0334 (0.9), 161.0236 (1.4), 135.0438 (0.9), 111.0436 (0.8), 93.0330 (2.5), 85.0279 (7.0)	3.20	0.070	B	1.22
90	coumaric acid-*O-*hexoside	C_15_H_18_O_8_	-	-	325.0930	325.0927 (3.8), 205.0501 (0.2), 163.0389 (100), 145.0282(13.3), 119.0488 (81.7)	3.36	−0.584	D1	0.77
91	(glucosyloxy-methoxyphenyl)-propanoic acid	C_16_H_22_O_9_	-	-	357.1191	357.1194 (6.4), 195.0654 (100), 151.0753 (2.3), 136.0517 (6.4), 123.0442 (1.6)	3.41	0.275	D1	8.95
92	caffeic acid ^a^	C_9_H_8_O_4_	-	-	179.0339	179.0340 (21.7), 135.0438 (100), 107.0488 (1.2)	3.56	−5.485	B	0.18
93	ferulic acid O-hexoside 2	C_16_H_20_O_9_	-	-	355.1035	355.1036 (8.4), 193.0497 (100), 178.0261 (16.2), 149.0596 (12.3), 134.0360 (38.1)	3.78	0.407	D1	1.07
94	*o-*coumaric acid ^a^	C_9_H_8_O_3_	-	-	163.0389	163.0389 (10.9), 119.0488 (100), 93.0330 (1.6)	4.57	−7.160	B	1.22
95	3, 5-dicaffeoylquinic acid	C_25_H_24_O_12_	-	-	515.1195	515.1192 (25.1), 353.0877 (94.7), 191.0553 (100), 179.0340 (51.0), 173.0446 (4.9), 135.0437 (51.5),	5.87	−0.581	D1	0.28
96	salicylic acid ^a^	C_7_H_6_O_3_	-	-	137.0230	137.0230(9.6), 108.0202 (3.3), 93.0330 (100)	6.26	−10.344	B	11.57
97	umbelliferone	C_9_H_6_O_3_	163.0390	163.0388 (100), 145.0283 (36.6), 135.0440 (60.5), 117.0337 (31.3), 107.0495 (8.6), 89.0391 (37.5)			6.26	−10.344	B	1.42
98	herniarin	C_10_H_8_O_3_	177.0546	177.0546 (46.3), 149.0596 (13.7), 145.0283 (100), 117.0337 (46.0), 89.0391 (27.8)			3.60	−0.117	D1	38.79

^a^: compared to reference standards; Confidence level: B: confirmed structure except for one or more stereochemical aspects; D: tentative identification based on libraries, model compounds, etc.; D1: relatively reliable evidence; * relative contents are expressed as % peak area of each compound to the total peak areas of the corresponding group secondary metabolites.

**Table 2:** Total bioactive components in the tested extract *.

Total Bioactive Compounds	
Total phenolic content (mg GAE/g)	27.7 ± 0.4
Total flavonoid content (mg RE/g)	31.6 ± 0.1

* Results are reported as mean ± SD of three parallel measurements. GAE: Gallic acid equivalent; RE: Rutin equivalent.

**Table 3:** Antioxidant properties of the tested extract and standards (IC_50_ and EC_50_ values (mg/mL)) *.

	DPPH Scavenging Ability	ABTS Scavenging Ability	CUPRAC	FRAP	Metal Chelating	Phosphomolybdenum
Extract	2.37 ± 0.02	1.21 ± 0.02	1.40 ± 0.01	0.61 ± 0.01	1.54 ± 0.06	1.96 ± 0.11
Trolox	0.05 ± 0.001	0.07 ± 0.001	0.14 ± 0.001	0.04 ± 0.001	-	0.49 ± 0.03
EDTA	-	-	-	-	0.02 ± 0.001	-

* Results are reported as mean ± SD of three parallel measurements.

**Table 4:** Enzyme inhibitory effects of the tested extract and standards (IC_50_ value (mg/mL)).

	AChE Inhibition	BChE Inhibition	Tyrosinase Inhibition	Amylase Inhibition	Glucosidase Inhibition
Extract	1.45 ± 0.01	ni	1.42 ± 0.02	4.02 ± 0.06	1.03 ± 0.02
Galanthamine	0.003 ± 0.0001	0.004 ± 0.0001	-	-	-
Kojic acid	-	-	0.08 ± 0.01	-	-
Acarbose	-	-	-	0.91 ± 0.01	1.07 ± 0.03

Results are reported as mean ± SD of three parallel measurements. ni: No inhibition observed at test concentrations up to 5 mg/mL.

**Table 5:** IC_50_ values (µg/mL) of plant extracts and standard compounds against enzymes associated with skin barrier support.

	Collagenase Inhibition	Elastase Inhibition	Hyaluronidase Inhibition
Extract	37.2 ± 2.0	39.8 ± 2.8	59.4 ± 5.5
EGCG	29.7 ± 2.11	30.83 ± 1.69	-
Tannic acid	-	-	36.93 ± 4.26

## Data Availability

The original contributions presented in this study are included in the article/Supplementary Material. Further inquiries can be directed to the corresponding author.

## Supplementary Materials

The following supporting information can be downloaded at https://www.mdpi.com/article/10.3390/plants15192918/s1: Figure S1: Extracted ion chromatogram of *S. perennis* flavonoids in negative ion mode (for numbers and fragmentation patterns, see Table 1); Figure S2: Extracted ion chromatogram of *S. perennis* maltol glycosides in positive ion mode (for numbers and fragmentation patterns, see Table 1); Figure S3: Extracted ion chromatogram of *S. perennis* phenolic acids and derivatives in negative ion mode (for numbers and fragmentation patterns, see Table 1) [40,41,42,43,44,45,46,47,48,49,50,51,52].

## References

[1] Smissen R.D., Garnock-Jones P.J. (2002). Relationships, classification and evolution of Scleranthus (Caryophyllaceae) as inferred from analysis of morphological characters. Bot. J. Linn. Soc..

[2] Greenberg A.K., Donoghue M.J. (2011). Molecular systematics and character evolution in Caryophyllaceae. TAXON.

[3] Iamonico D. (2023). Maguirellaria (Caryophyllaceae), a new genus from Dominican Republic. Phytotaxa.

[4] Jakimiuk K., Strawa J.W., Granica S., Locatelli M., Tartaglia A., Tomczyk M. (2022). Determination of Flavonoids in Selected Scleranthus Species and Their Anti-Collagenase and Antioxidant Potential. Molecules.

[5] Jakimiuk K., Strawa J.W., Granica S., Tomczyk M. (2021). New Flavone C-Glycosides from *Scleranthus perennis* and Their Anti-Collagenase Activity. Molecules.

[6] Yayli N., Seymen H., Baltaci C. (2001). Flavone C-glycosides from *Scleranthus uncinatus*. Phytochemistry.

[7] Jakimiuk K., Wink M., Tomczyk M. (2022). Flavonoids of the Caryophyllaceae. Phytochem. Rev..

[8] Cheikh-Ali S., Farman M., Lacaille-Dubois M.-A., Semmar N. (2019). Structural organization of saponins in Caryophyllaceae. Phytochem. Rev..

[9] Gevrenova R., Zaharieva M.M., Kroumov A.D., Voutquenne-Nazabadioko L., Zheleva-Dimitrova D., Balabanova V., Hajdenski H.M., Konstantinov S. (2019). Gypsophila saponins enhance the cytotoxicity of etoposide in HD-MY-Z lymphoma cells. Food Chem. Toxicol..

[10] Gevrenova R., Bardarov K., Bouguet-Bonnet S., Voynikov Y., Balabanova V., Zheleva-Dimitrova D., Henry M. (2018). A new liquid chromatography-high resolution Orbitrap mass spectrometry-based strategy to characterize Glucuronide Oleanane-type Triterpenoid Carboxylic Acid 3, 28-O-Bidesmosides (GOTCAB) saponins. A case study of *Gypsophila glomerata* Pall ex M. B. (Caryophyllaceae). J. Pharm. Biomed. Anal..

[11] Neve C., Gevrenova R., Sayagh C., Martinez A., Abou Dib A., Voutquenne-Nazabadioko L. (2026). Triterpenoid saponins from the roots of *Gypsophila perfoliata* L.. Phytochemistry.

[12] Gevrenova R., Nawrot-Hadzik I., Matkowski A. (2025). Oleanane-type bidesmosides from *Agrostemma githago* L. roots and aerial parts. Biochem. Syst. Ecol..

[13] Çiçek S.S., Mangoni A., Hanschen F.S., Agerbirk N., Zidorn C. (2024). Essentials in the acquisition, interpretation, and reporting of plant metabolite profiles. Phytochemistry.

[14] Ferreres F., Gil-Izquierdo A., Andrade P.B., Valentão P., Tomás-Barberán F. (2007). Characterization of C-glycosyl flavones O-glycosylated by liquid chromatography–tandem mass spectrometry. J. Chromatogr. A.

[15] Sinan K.I., Zengin G., Zheleva-Dimitrova D., Gevrenova R., Picot-Allain M.C.N., Dall’Acqua S., Behl T., Goh B.H., Ying P.T.S., Mahomoodally M.F. (2021). Exploring the chemical profiles and biological values of two Spondias species (*S. dulcis* and *S. mombin*): Valuable Sources of Bioactive Natural Products. Antioxidants.

[16] Zhang Y., Qu L., Liu L., Li X., Liu E., Han L., Fang S., Gao X., Wang T. (2015). New maltol glycosides from Flos Sophorae. J. Nat. Med..

[17] Kozachok S., Pecio Ł., Kolodziejczyk-Czepas J., Marchyshyn S., Nowak P., Mołdoch J., Oleszek W. (2018). γ-Pyrone compounds: Flavonoids and maltol glucoside derivatives from *Herniaria glabra* L. collected in the Ternopil region of the Ukraine. Phytochemistry.

[18] Yayli N., Baltac C., Genç H., Terzioglu S. (2002). Phenolic and Flavone C-Glycosides from *Scleranthus uncinatus*. Pharm. Biol..

[19] Kozachok S., Kolodziejczyk-Czepas J., Marchyshyn S., Wojtanowski K.K., Zgórka G., Oleszek W. (2022). Comparison of Phenolic Metabolites in Purified Extracts of Three Wild-Growing *Herniaria* L. Species and Their Antioxidant and Anti-Inflammatory Activities In Vitro. Molecules.

[20] Yang J., Guo J., Yuan J. (2008). In vitro antioxidant properties of rutin. LWT-Food Sci. Technol..

[21] Wang Q., Wei H.C., Zhou S.J., Li Y., Zheng T.T., Zhou C.Z., Wan X.H. (2022). Hyperoside: A review on its sources, biological activities, and molecular mechanisms. Phytother. Res..

[22] Ahmadi S.M., Farhoosh R., Sharif A., Rezaie M. (2020). Structure-antioxidant activity relationships of luteolin and catechin. J. Food Sci..

[23] Xie L., Deng Z., Zhang J., Dong H., Wang W., Xing B., Liu X. (2022). Comparison of Flavonoid O-Glycoside, C-Glycoside and Their Aglycones on Antioxidant Capacity and Metabolism during In Vitro Digestion and In Vivo. Foods.

[24] Zielińska D., Zieliński H. (2025). Multifaceted Biological Activity of Selected Flavone C-Monoglucosides. Int. J. Mol. Sci..

[25] Chinnapun D. (2025). The Structure and Biological Activities With Health Benefits of Luteolin Glycosides From Plants. Sci. World J..

[26] Ghozzi I., Fontaine J.-X., Molinié R., Elboutachfaiti R., Akkouche L., Sebei K., Mathiron D., Hano C., Garros L., Choque E. (2024). Relationship Between the Structure of the Flavone C-Glycosides of Linseed (*Linum usitatissimum* L.) and Their Antioxidant Activity. Molecules.

[27] Singh K., Bhushan B., Mittal N., Kushwaha A., Raikwar C.K., Sharma A.K., Chanchal D.K., Kumar S., Agrawal M. (2024). Recent Advances in Enzyme Inhibition: A Pharmacological Review. Curr. Enzym. Inhib..

[28] Ertürk A., Gulcin I. (2026). Deciphering the Antioxidant Activity and Enzyme Inhibition of Luteolin and Its Glycosides: An Integrated In Vitro and In Silico Approach. Catalysts.

[29] Li Y.Q., Zhou F.C., Gao F., Bian J.S., Shan F. (2009). Comparative evaluation of quercetin, isoquercetin and rutin as inhibitors of α-glucosidase. J. Agric. Food Chem..

[30] Zhu J., Chen C., Zhang B., Huang Q. (2020). The inhibitory effects of flavonoids on α-amylase and α-glucosidase. Crit. Rev. Food Sci. Nutr..

[31] Jakimiuk K., Sari S., Milewski R., Supuran C.T., Şöhretoğlu D., Tomczyk M. (2022). Flavonoids as tyrosinase inhibitors in in silico and in vitro models: Basic framework of SAR using a statistical modelling approach. J. Enzym. Inhib. Med. Chem..

[32] Şöhretoğlu D., Sari S. (2020). Flavonoids as alpha-glucosidase inhibitors: Mechanistic approaches merged with enzyme kinetics and molecular modelling. Phytochem. Rev..

[33] Chang C.C., Ho S.L., Lee S.S. (2015). Acylated glucosylflavones as α-glucosidase inhibitors from *Tinospora crispa* leaf. Bioorg. Med. Chem..

[34] Costa G.M., Cárdenas P.A., Gazola A.C., Aragón D.M., Castellanos L., Reginatto F.H., Ramos F.A., Schenkel E.P. (2015). Isolation of C-glycosylflavonoids with α-glucosidase inhibitory activity from *Passiflora bogotensis* Benth by gradient high-speed counter-current chromatography. J. Chromatogr. B Anal. Technol. Biomed. Life Sci..

[35] An Online Flora of All Known Plants. https://www.worldfloraonline.org.

[36] Zheleva-Dimitrova D., Gevrenova R., Zaharieva M.M., Najdenski H., Ruseva S., Lozanov V., Balabanova V., Yagi S., Momekov G., Mitev V. (2017). HPLC-UV and LC–MS Analyses of Acylquinic Acids in *Geigeria alata* (DC) Oliv. & Hiern. and their Contribution to Antioxidant and Antimicrobial Capacity. Phytochem. Anal..

[37] Gevrenova R., Zengin G., Sinan K.I., Zheleva-Dimitrova D., Balabanova V., Kolmayer M., Voynikov Y., Joubert O. (2023). An In-Depth Study of Metabolite Profile and Biological Potential of *Tanacetum balsamita* L. (Costmary). Plants.

[38] Yagi S., Zengin G., Uba A.I., Maciejewska-Turska M., Sieniawska E., Świątek Ł., Rajtar B., Bahşi M., Guler O., Dall’Acqua S. (2024). Exploring chemical composition, antioxidant, enzyme inhibitory and cytotoxic properties of *Glaucium acutidentatum* Hausskn. & Bornm. from Turkey Flora: A novel source of bioactive agents to design functional applications. Antioxidants.

[39] Yıldırım A., Celep İ.K., Gül A. (2025). Cosmetological potential of the essential oil from the aerial parts of *Rubus sanctus* Schreb.: Enzymatic inhibition, antibacterial properties, and chemical composition. J. Essent. Oil Bear. Plants.

[40] Gevrenova R., Szakiel A., Pączkowski C., Zengin G., Kurt-Celep I., Stefanova A., Zheleva-Dimitrova D. (2025). *Erica spiculifolia* Salisb. (Balkan Heath): A Focus on Metabolic Profiling and Antioxidant and Enzyme Inhibitory Properties. Plants.

[41] Slinkard K., Singleton V.L. (1977). Total phenol analysis: Automation and comparison with manual methods. Am. J. Enol. Vitic..

[42] Arvouet-Grand A., Vennat B., Pourrat A., Legret P. (1994). Standardization of propolis extract and identification of principal constituents. J. Pharm. Belg..

[43] Kirby A.J., Schmidt R.J. (1997). The antioxidant activity of Chinese herbs for eczema and of placebo herbs—I. J. Ethnopharmacol..

[44] Re R., Pellegrini N., Proteggente A., Pannala A., Yang M., Rice-Evans C. (1999). Antioxidant activity applying an improved ABTS radical cation decolorization assay. Free Radic. Biol. Med..

[45] Apak R., Güçlü K., Özyürek M., Karademir S.E. (2004). Novel total antioxidant capacity index for dietary polyphenols and vitamins C and E, using their cupric ion reducing capability in the presence of neocuproine: CUPRAC method. J. Agric. Food Chem..

[46] Benzie I.F., Strain J.J. (1996). The ferric reducing ability of plasma (FRAP) as a measure of “antioxidant power”: The FRAP assay. Anal. Biochem..

[47] Prieto P., Pineda M., Aguilar M. (1999). Spectrophotometric quantitation of antioxidant capacity through the formation of a phosphomolybdenum complex: Specific application to the determination of vitamin E. Anal. Biochem..

[48] Dinis T.C., Madeira V.M., Almeida L.M. (1994). Action of phenolic derivatives (acetaminophen, salicylate, and 5-aminosalicylate) as inhibitors of membrane lipid peroxidation and as peroxyl radical scavengers. Arch. Biochem. Biophys..

[49] Ellman G.L., Courtney K.D., Andres V., Featherstone R.M. (1961). A new and rapid colorimetric determination of acetylcholinesterase activity. Biochem. Pharmacol..

[50] Masuda T., Yamashita D., Takeda Y., Yonemori S. (2005). Screening for tyrosinase inhibitors among extracts of seashore plants and identification of potent inhibitors from Garcinia subelliptica. Biosci. Biotechnol. Biochem..

[51] Šafašík I. (1990). Rapid Detection of Alpha-Amylase Inhibitors. J. Enzym. Inhib..

[52] Ting L., Zhang X.-D., Song Y.-W., Liu J.-W. (2005). A microplate-based screening method for alpha-glucosidase inhibitors. Chin. J. Clin. Pharmacol. Ther..

