# Supplementary material for: Scleranthus perennis L.: Towards Comprehensive Metabolite Profiling, Antioxidant and Enzyme Inhibitory Activity
Source: Plants (Basel). 2026 Sep 23;15(19):2918. doi: 10.3390/plants15192918 (PMC13666577; doi:10.3390/plants15192918)
Supplement: Supplementary file 1 [file plants-15-02918-s001.zip › plants-4524182-supplementary.pdf]

Scleranthus perennis L.: Towards comprehensive metabolite profiling, antioxidant and enzyme inhibitory activity

Reneta Gevrenova[a,*], Inci Kurt-Celep[b], Gokhan Zengin[c], Dimitrina Zheleva-Dimitrova[a]

[a]Department of Pharmacognosy, Faculty of Pharmacy, Medical University, 1000 Sofia, Bulgaria
[b]Department of Pharmaceutical Biotechnology, Faculty of Pharmacy, Istanbul Okan University, Tuzla, Istanbul 34940, Turkey, inci.celep@okan.edu.tr (IKC)
[c]Physiology and Biochemistry Research Laboratory, Department of Biology, Science Faculty, Selcuk University, Konya 42130, Turkey, biyologzengin@gmail.com (GZ)
* Correspondence: rgevrenova@pharmfac.mu-sofia.bg (R. Gevrenova)

Supplemental material

Assays for Total Phenolic and Flavonoid Contents

The total phenolic content was determined by employing the methods given in the literature with some modification. Sample solution (0.25 mL) was mixed with diluted Folin–Ciocalteu reagent (1 mL, 1:9, v/v) and shaken vigorously. After 3 min, Na_2CO_3 solution (0.75 mL, 1%) was added and the sample absorbance was read at 760 nm after a 2 h incubation at room temperature. The total phenolic content was expressed as milligrams of gallic acid equivalents (mg GAE/g extract) [1]

The total flavonoid content was determined using the $AlCl_3$ method. Briefly, sample solution (1 mL) was mixed with the same volume of aluminum trichloride (2%) in methanol. Similarly, a blank was prepared by adding sample solution (1 mL) to methanol (1 mL) without $AlCl_3$. The sample and blank absorbances were read at 415 nm after a 10 min incubation at room temperature. The absorbance of the blank was subtracted from that of the sample. Rutin was used as a reference standard and the total flavonoid content was expressed as milligrams of rutin equivalents (mg RE/g extract) [2]

Determination of Antioxidant and Enzyme Inhibitory Effects

Antioxidant (DPPH and ABTS radical scavenging, reducing power (CUPRAC and FRAP), phosphomolybdenum and metal chelating (ferrozine method)) and enzyme inhibitory activities (cholinesterase (Elmann's method), tyrosinase (dopachrome method), α-amylase (iodine/potassium iodide method), α -glucosidase (chromogenic PNPG method) and pancreatic lipase (*p*-nitrophenyl butyrate (p-NPB) method) were determined.

For the DPPH (1,1-diphenyl-2-picrylhydrazyl) radical scavenging assay: Sample solution was added to 4 mL of a 0.004% methanol solution of DPPH. The sample absorbance was read at 517 nm after a 30 min incubation at room temperature in the dark. Trolox was used as a positive control. DPPH radical scavenging activity was expressed as IC_{50} value (mg/ml) [3].

For ABTS (2,2′-azino-bis(3-ethylbenzothiazoline) 6-sulfonic acid) radical scavenging assay: Briefly, ABTS+ was produced directly by reacting 7 mM ABTS solution with 2.45 mM potassium persulfate and allowing the mixture to stand for 12–16 in the dark at room temperature. Prior to beginning the assay, ABTS

solution was diluted with methanol to an absorbance of 0.700 ± 0.02 at 734 nm. Sample solution was added to ABTS solution (2 mL) and mixed. The sample absorbance was read at 734 nm after a 30 min incubation at room temperature. Trolox was used as a positive control. The ABTS radical scavenging activity was expressed as IC_{50} value (mg/ml) [4].

For CUPRAC (cupric ion reducing activity) activity assay: Sample solution was added to premixed reaction mixture containing $CuCl_2$ (1 mL, 10 mM), neocuproine (1 mL, 7.5 mM) and NH_4Ac buffer (1 mL, 1 M, pH 7.0). Similarly, a blank was prepared by adding sample solution (0.5 mL) to premixed reaction mixture (3 mL) without $CuCl_2$. Then, the sample and blank absorbances were read at 450 nm after a 30 min incubation at room temperature. The absorbance of the blank was subtracted from that of the sample. Trolox was used as a positive control. CUPRAC activity was expressed as EC_{50} value (effective concentration at absorbace 0.5) (mg/ml) [5].

For FRAP (ferric reducing antioxidant power) activity assay: Sample solution was added to premixed FRAP reagent (2 mL) containing acetate buffer (0.3 M, pH 3.6), 2,4,6-tris(2-pyridyl)-S-triazine (TPTZ) (10 mM) in 40 mM HCl and ferric chloride (20 mM) in a ratio of 10:1:1 (v/v/v). Then, the sample absorbance was read at 593 nm after a 30 min incubation at room temperature. Trolox was used as a positive control. FRAP activity was expressed as as EC_{50} value (mg/ml) [6].

For phosphomolybdenum method: Sample solution was combined with 3 mL of reagent solution (0.6 M sulfuric acid, 28 mM sodium phosphate and 4 mM ammonium molybdate). The sample absorbance was read at 695 nm after a 90 min incubation at 95 °C. Trolox was used as a positive control. The total antioxidant capacity was expressed as EC_{50} value (mg/ml) [7].

For metal chelating activity assay: Briefly, sample solution was added to $FeCl_2$ solution (0.05 mL, 2 mM). The reaction was initiated by the addition of 5 mM ferrozine (0.2 mL). Similarly, a blank was prepared by adding sample solution (2 mL) to $FeCl_2$ solution (0.05 mL, 2 mM) and water (0.2 mL) without ferrozine. Then, the sample and blank absorbances were read at 562 nm after 10 min incubation at room temperature. The absorbance of the blank was subtracted from that of the sample. EDTA was used as a positive control. The metal chelating activity was expressed as IC50 value (mg/ml).[8].

For Cholinesterase (ChE) inhibitory activity assay: Sample solution (was mixed with DTNB (5,5-dithio-bis(2-nitrobenzoic) acid, Sigma, St. Louis, MO, USA) (125 µL) and AChE (acetylcholinesterase (Electric ell acetylcholinesterase, Type-VI-S, EC 3.1.1.7,Sigma)), or BChE (butyrylcholinesterase (horse serum butyrylcholinesterase, EC 3.1.1.8, Sigma)) solution (25 µL) in Tris–HCl buffer (pH 8.0) in a 96-well microplate and incubated for 15 min at 25 °C. The reaction was then initiated with the addition of acetylthiocholine iodide (ATCI, Sigma) or butyrylthiocholine chloride (BTCl, Sigma) (25 µL). Similarly, a blank was prepared by adding sample solution to all reaction reagents without enzyme (AChE or BChE) solution. The sample and blank absorbances were read at 405 nm after 10 min incubation at 25 °C. Galantamine was used as a standard inhibitor. The absorbance of the blank was subtracted from that of the sample and the cholinesterase inhibitory activity was expressed as as IC_{50} value (mg/ml) [9].

For Tyrosinase inhibitory activity assay: Sample solution was mixed with tyrosinase solution (40 µL, Sigma) and phosphate buffer (100 µL, pH 6.8) in a 96-well microplate and incubated for 15 min at 25 °C.

The reaction was then initiated with the addition of L-DOPA (40 μL, Sigma). Similarly, a blank was prepared by adding sample solution to all reaction reagents without enzyme (tyrosinase) solution. The sample and blank absorbances were read at 492 nm after a 10 min incubation at 25 °C. Kojic acid was used as a standard inhibitor .The absorbance of the blank was subtracted from that of the sample and the tyrosinase inhibitory activity was expressed as IC_{50} value (mg/ml) [10].

For α-amylase inhibitory activity assay: Sample solution was mixed with α-amylase solution (ex-porcine pancreas, EC 3.2.1.1, Sigma) (50 μL) in phosphate buffer (pH 6.9 with 6 mM sodium chloride) in a 96-well microplate and incubated for 10 min at 37 °C. After pre-incubation, the reaction was initiated with the addition of starch solution (50 μL, 0.05%). Similarly, a blank was prepared by adding sample solution to all reaction reagents without enzyme (α-amylase) solution. The reaction mixture was incubated 10 min at 37 °C. The reaction was then stopped with the addition of HCl (25 μL, 1 M). This was followed by addition of the iodine-potassium iodide solution (100 μL). The sample and blank absorbances were read at 630 nm. Acarbose was used as a standard inhibitor. The absorbance of the blank was subtracted from that of the sample and the α-amylase inhibitory activity was expressed as IC_{50} value (mg/ml) [11].

For α-glucosidase inhibitory activity assay: Sample solution was mixed with glutathione (50 μL), α-glucosidase solution (from Saccharomyces cerevisiae, EC 3.2.1.20, Sigma) (50 μL) in phosphate buffer (pH 6.8) and PNPG (4-N-trophenyl-α-D-glucopyranoside, Sigma) (50 μL) in a 96-well microplate and incubated for 15 min at 37 °C. Similarly, a blank was prepared by adding sample solution to all reaction reagents without enzyme (α-glucosidase) solution. The reaction was then stopped with the addition of sodium carbonate (50 μL, 0.2 M). The sample and blank absorbances were read at 400 nm. Acarbose was used as a standard inhibitor. The absorbance of the blank was subtracted from that of the sample and the α-glucosidase inhibitory activity was expressed as IC_{50} value (mg/ml) [12].

Collagenase from *Clostridium histolyticum* (EC 3.4.24.3) and the synthetic substrate FALGPA (N-[3-(2-furyl)acryloyl]-Leu-Gly-Pro-Ala) were used to assess the inhibitory action of collagenase. The 50 mM Tricine buffer at pH 7.5 made up the test medium. For each test, 80 μL of buffer, 10 μL of FALGPA (1 mM), and 10 μL of collagenase solution (0.8 U/mL) were combined to create a total reaction volume. 10 μL of plant extract, varying in concentration from 62.5 to 1000 μg/mL, was added to this mixture for the test samples. The decrease in absorbance at 340 nm, which indicates the substrate breakdown, was used to measure the enzymatic activity following a 30-minute incubation period at 25°C. In this experiment, the positive control was epigallocatechin gallate (EGCG) [13, 14]. The results were expressed as IC50 value (μg/ml).

Elastase inhibition studies were carried out using elastase enzyme obtained from porcine pancreas (1 U/mL) and N-Succinyl-Ala-Ala-Ala-p-nitroanilide substrate. The reaction mixture was prepared in 50 mM Tris-HCl buffer (pH 8.0). For each sample, 50 μL of enzyme solution, 25 μL of test sample (plant extract or control solution), and 100 μL of buffer solution were combined. The mixtures were incubated at room temperature for 10 min, and then 25 μL of substrate solution was added to initiate the reaction. The amount of p-nitroaniline formed by the elastase enzyme by cleaving the substrate was measured spectrophotometrically at 405 nm. Plant extracts were applied at increasing concentrations ranging from 62.5–1000 μg/mL and the percentage of elastase inhibition at each concentration was calculated compared

to the control group. Based on the obtained data, a dose-response curve was constructed and the IC_{50} value was determined. EGCG was used as positive control [13, 14]. The results were expressed as IC50 value (μg/ml).

Hyaluronidase inhibition study was performed using commercially available hyaluronidase enzyme (EC 3.2.1.35, Sigma-Aldrich). The enzyme was dissolved in 0.02 M phosphate buffer (pH 3.5) containing NaCl and BSA. Hyaluronic acid substrate was prepared in 0.1 M acetate buffer. The reaction mixture was composed of 20 μL of plant extract at increasing concentrations (62.5-1000 μg/mL), 10 μL of enzyme solution, and 60 μL of acetate buffer, and the mixture was incubated at 37°C for 20 minutes. Then, 10 μL of substrate was added to initiate the reaction and incubated for a second time for 20 minutes. At the end of the reaction, the inhibition rate was calculated by measuring the absorbance at 600 nm. Plant extracts were applied at different concentrations, % inhibition values were evaluated by comparing with the control group, and IC_{50} values were determined from the dose-response curve. Tannic acid was used as the standard inhibitor, and comparison was made with the reference IC_{50} value.

References

1. Slinkard, K.; Singleton, V. L., Total phenol analysis: automation and comparison with manual methods. *American journal of enology and viticulture* **1977,** 28, (1), 49–55.

2. Arvouet-Grand, A.; Vennat, B.; Pourrat, A.; Legret, P., [Standardization of propolis extract and identification of principal constituents]. *J Pharm Belg* **1994,** 49, (6), 462–8.

3. Kirby, A. J.; Schmidt, R. J., The antioxidant activity of Chinese herbs for eczema and of placebo herbs—I. *Journal of ethnopharmacology* **1997,** 56, (2), 103–108.

4. Re, R.; Pellegrini, N.; Proteggente, A.; Pannala, A.; Yang, M.; Rice-Evans, C., Antioxidant activity applying an improved ABTS radical cation decolorization assay. *Free radical biology and medicine* **1999,** 26, (9-10), 1231–1237.

5. Apak, R.; Güçlü, K.; Özyürek, M.; Karademir, S. E., Novel total antioxidant capacity index for dietary polyphenols and vitamins C and E, using their cupric ion reducing capability in the presence of neocuproine: CUPRAC method. *Journal of agricultural and food chemistry* **2004,** 52, (26), 7970–7981.

6. Benzie, I. F.; Strain, J. J., The ferric reducing ability of plasma (FRAP) as a measure of "antioxidant power": the FRAP assay. *Analytical biochemistry* **1996,** 239, (1), 70–76.

7. Prieto, P.; Pineda, M.; Aguilar, M., Spectrophotometric quantitation of antioxidant capacity through the formation of a phosphomolybdenum complex: specific application to the determination of vitamin E. *Analytical biochemistry* **1999,** 269, (2), 337–341.

8. Dinis, T. C.; Madeira, V. M.; Almeida, L. M., Action of phenolic derivatives (acetaminophen, salicylate, and 5-aminosalicylate) as inhibitors of membrane lipid peroxidation and as peroxyl radical scavengers. *Archives of biochemistry and biophysics* **1994,** 315, (1), 161–169.

9. Ellman, G. L.; Courtney, K. D.; Andres Jr, V.; Featherstone, R. M., A new and rapid colorimetric determination of acetylcholinesterase activity. *Biochemical pharmacology* **1961,** 7, (2), 88–95.

10. Masuda, T.; Yamashita, D.; Takeda, Y.; Yonemori, S., Screening for tyrosinase inhibitors among extracts of seashore plants and identification of potent inhibitors from Garcinia subelliptica. *Bioscience, biotechnology, and biochemistry* **2005,** 69, (1), 197–201.

11. Šafašík, I., Rapid Detection of Alpha-Amylase Inhibitors. *Journal of Enzyme Inhibition* **1990,** 3, (3), 245–247.

12. Ting, L.; Zhang, X.-d.; Song, Y.-w.; Liu, J.-w., A microplate-based screening method for alpha-glucosidase inhibitors. *Chinese Journal of Clinical Pharmacology and Therapeutics* **2005,** 10, (10), 1128.

13. Yıldırım, A.; Celep, İ. K.; Gül, A., Cosmetological potential of the essential oil from the aerial parts of Rubus sanctus Schreb.: Enzymatic inhibition, antibacterial properties, and chemical composition. *Journal of Essential Oil Bearing Plants* **2025,** 28, (3), 471–486.

14. Gevrenova, R.; Szakiel, A.; Pączkowski, C.; Zengin, G.; Kurt-Celep, I.; Stefanova, A.; Zheleva-Dimitrova, D., Erica spiculifolia Salisb. (Balkan Heath): A Focus on Metabolic Profiling and Antioxidant and Enzyme Inhibitory Properties. *Plants* **2025,** 14, (11), 1648.

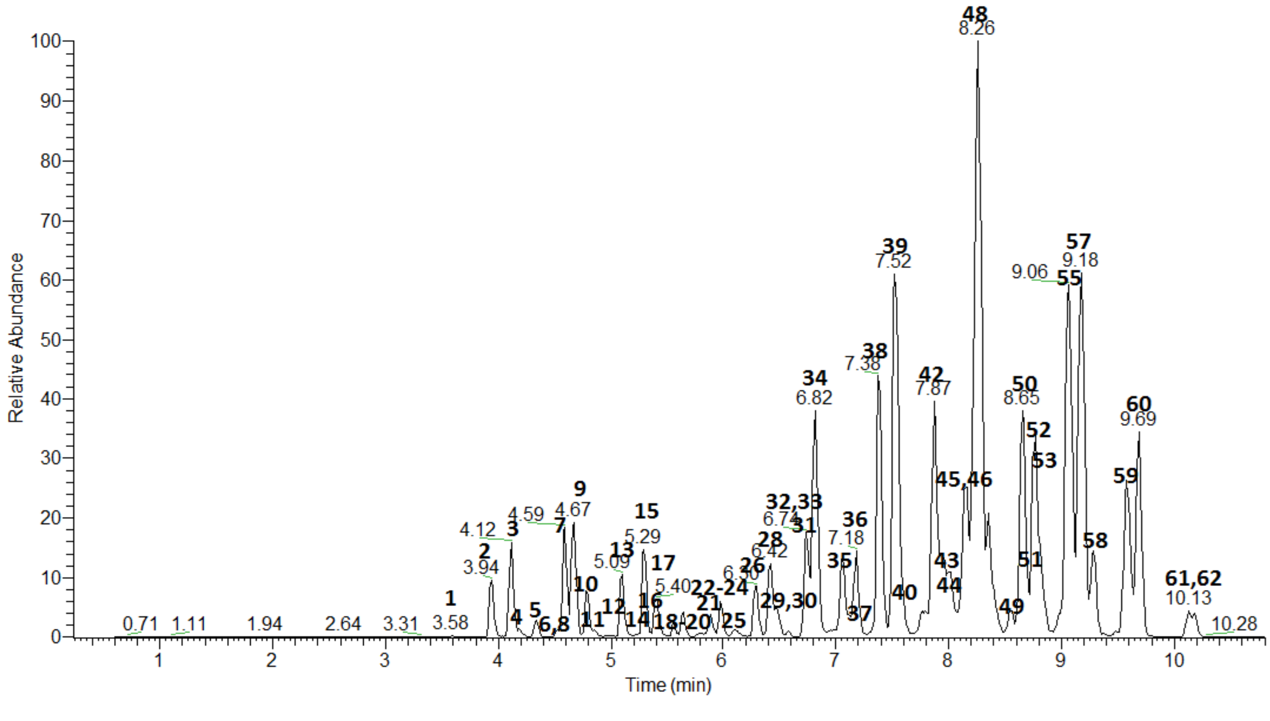

Figure S1. Extracted ion chromatogram of *S. perennis* flavonoids in negative ion mode (for numbers and fragmentation patterns, see Table 1).

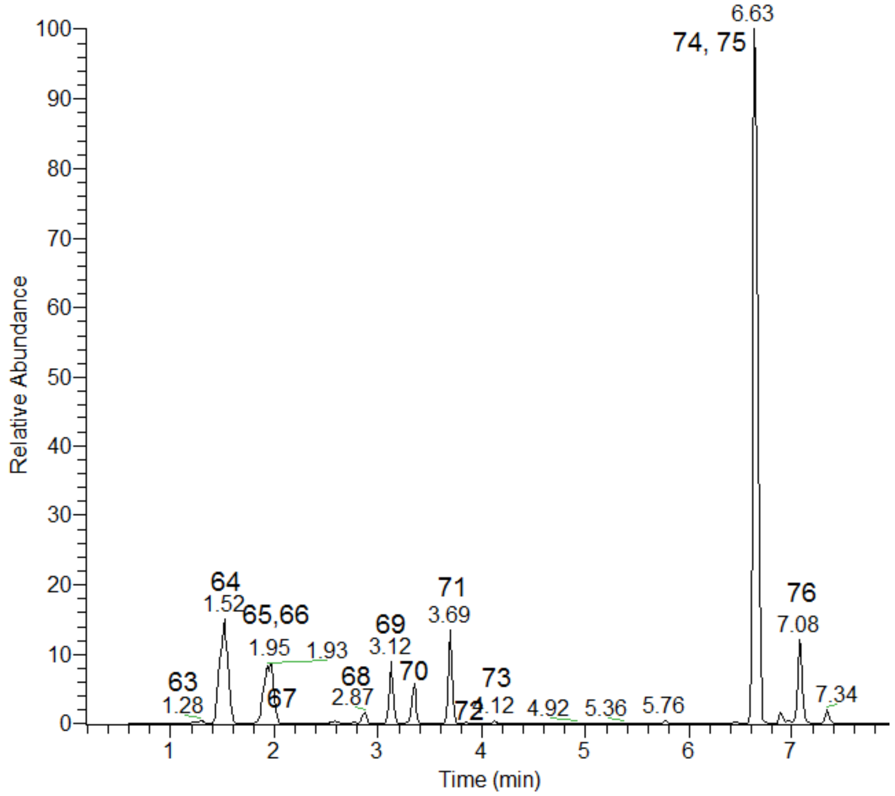

Figure S2. Extracted ion chromatogram of *S. perennis* maltol glycosides in positive ion mode (for numbers and fragmentation patterns, see Table 1).

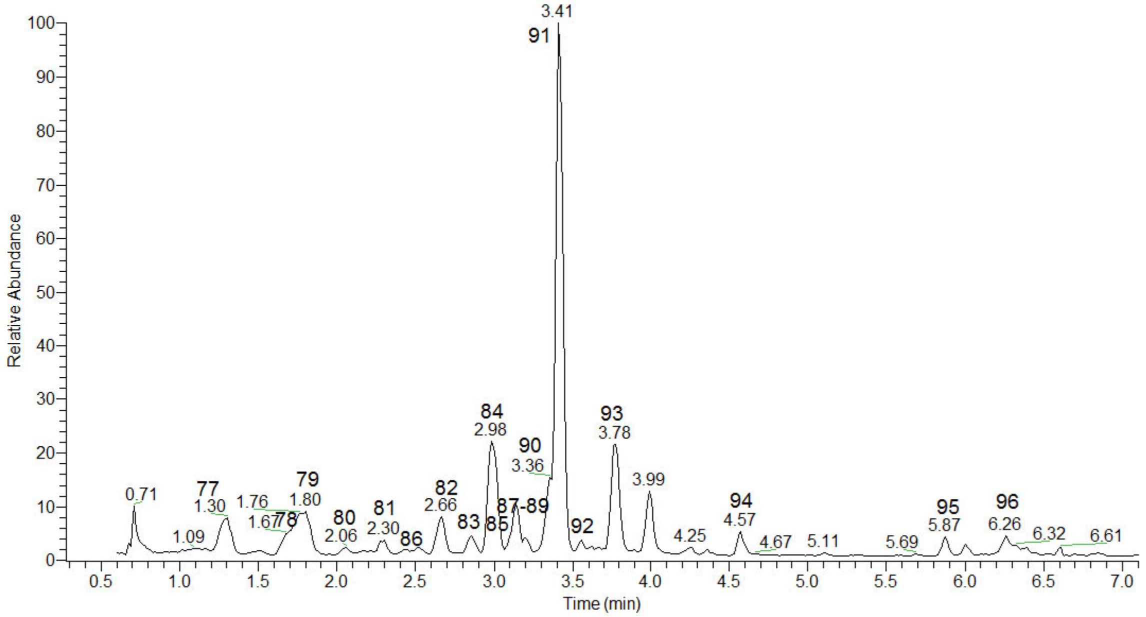

Figure S3. Extracted ion chromatogram of *S. perennis* phenolic acids and derivatives in negative ion mode (for numbers and fragmentation patterns, see Table 1).
